# Biomarkers of Tolerance in Kidney Transplantation: Are We Predicting Tolerance or Response to Immunosuppressive Treatment?

**DOI:** 10.1111/ajt.13932

**Published:** 2016-08-08

**Authors:** I. Rebollo‐Mesa, E. Nova‐Lamperti, P. Mobillo, M. Runglall, S. Christakoudi, S. Norris, N. Smallcombe, Y. Kamra, R. Hilton, Indices of Tolerance EU Consortium, S. Bhandari, R. Baker, D. Berglund, S. Carr, D. Game, S. Griffin, P. A. Kalra, R. Lewis, P. B. Mark, S. Marks, I. Macphee, W. McKane, M. G. Mohaupt, R. Pararajasingam, S. P. Kon, D. Serón, M. D. Sinha, B. Tucker, O. Viklický, R. I. Lechler, G. M. Lord, M. P. Hernandez‐Fuentes

**Affiliations:** ^1^ Medical Research Council Centre for TransplantationKing's College LondonLondonUnited Kingdom; ^2^ BiostatisticsInstitute of Psychiatry, Psychology and NeuroscienceKing's College LondonLondonUnited Kingdom; ^3^ UCB CelltechUCB Pharma S.A.SloughUnited Kingdom; ^4^ National Institute for Health Research Biomedical Research CentreGuy's and St. Thomas’ National Health Service Foundation TrustKing's College LondonLondonUnited Kingdom; ^5^ University College LondonLondonUnited Kingdom; ^6^ Peter Gorer Department of ImmunobiologyKing's College LondonLondonUnited Kingdom; ^7^ Guy's and St. Thomas’ NHS Foundation TrustLondonUnited Kingdom; ^8^ King's College LondonLondonUnited Kingdom; ^9^ Oxford UniversityOxfordUnited Kingdom; ^10^ Imperial College LondonLondonUnited Kingdom; ^11^ Institute for Medical Immunology, Université Libre de BruxellesBruxellesBelgium; ^12^ Miltenyi BiotecBergisch GladbachGermany; ^13^ University of NantesNantesFrance; ^14^ Charité, Universitaatsmedizin BerlinBerlinGermany; ^15^ Hull and East Yorkshire Hospitals NHS TrustHullUnited Kingdom; ^16^ St. James's University HospitalLeedsUnited Kingdom; ^17^ Uppsala University HospitalUppsalaSweden; ^18^ Leicester General HospitalLeicesterUnited Kingdom; ^19^ Cardiff and Vale University Health BoardCardiffUnited Kingdom; ^20^ Salford Royal HospitalSalfordUnited Kingdom; ^21^ Queen Alexandra HospitalPortsmouthUnited Kingdom; ^22^ University of GlasgowGlasgowUnited Kingdom; ^23^ Great Ormond Street Hospital for Children NHS Foundation TrustLondonUnited Kingdom; ^24^ St. George's HospitalLondonUnited Kingdom; ^25^ Northern General HospitalSheffieldUnited Kingdom; ^26^ INSELSPITALUniversitätsspital BernKlinik für Nephrologie/Hypertonie Abteilung für HypertonieBernSwitzerland; ^27^ Manchester Royal InfirmaryManchesterUnited Kingdom; ^28^ King's College Hospital NHS Foundation TrustLondonUnited Kingdom; ^29^ Hospital Universitari Vall d'Hebr_onBarcelonaSpain; ^30^ Evelina London Children's HospitalLondonUnited Kingdom; ^31^ Transplantační laboratoř IKEMPragueCzech Republic; ^32^ King's Health PartnersLondonUnited Kingdom

**Keywords:** translational research/science, kidney transplantation/nephrology, immunobiology, molecular biology, biomarker, kidney (allograft) function/dysfunction, tolerance

## Abstract

We and others have previously described signatures of tolerance in kidney transplantation showing the differential expression of B cell–related genes and the relative expansions of B cell subsets. However, in all of these studies, the index group—namely, the tolerant recipients—were not receiving immunosuppression (IS) treatment, unlike the rest of the comparator groups. We aimed to assess the confounding effect of these regimens and develop a novel IS‐independent signature of tolerance. Analyzing gene expression in three independent kidney transplant patient cohorts (232 recipients and 14 tolerant patients), we have established that the expression of the previously reported signature was biased by IS regimens, which also influenced transitional B cells. We have defined and validated a new gene expression signature that is independent of drug effects and also differentiates tolerant patients from healthy controls (cross‐validated area under the receiver operating characteristic curve [AUC] = 0.81). In a prospective cohort, we have demonstrated that the new signature remained stable before and after steroid withdrawal. In addition, we report on a validated and highly accurate gene expression signature that can be reliably used to identify patients suitable for IS reduction (approximately 12% of stable patients), irrespective of the IS drugs they are receiving. Only a similar approach will make the conduct of pilot clinical trials for IS minimization safe and hence allow critical improvements in kidney posttransplant management.

## Abstract 2

The relationship between B cell gene expression and transplantation tolerance is confounded by the effects of immunosuppressive drugs, and correcting for this effect on gene expression is possible, exposing an equally accurate signature of tolerance. See page 3320 for Markmann's editorial.

**Abbreviations**
AUC: area under the receiver operating characteristic curve
Aza: azathioprine
CNI: calcineurin inhibitor
Cyc: ciclosporin A
DMSO: dimethyl sulfosxide
GMCSF: granulocyte–macrophage colony‐stimulating factor
IS‐IE: IS‐independent expression, the new signature of tolerance
IS: immunosuppression
KTR: kidney transplant recipient
MMF: mycophenolate mofetil
NFκB: nuclear factor kappa B
PBMC: peripheral blood mononuclear cell
Pred: prednisone
REGGR: glucocorticoid receptor regulatory network
Tac: tacrolimus
TNF: tumor necrosis factor

## Introduction

Despite improvements in the stratification of therapy in kidney transplant recipients (KTRs), grafts do not display the desired longevity. The immunosuppression (IS) needs of individual patients are poorly defined, and the current approaches are far from an ideal personalized management program. Standard KTRs are maintained with calcineurin inhibitors (CNIs) or sirolimus on doses defined by blood levels and azathioprine (Aza) is adjusted on weight, whereas mycophenolate mofetil (MMF) or prednisone doses are given as population‐based results dictate 1, 2. As a result, overimmunosuppression is responsible for a high number of patients with a functioning graft dying or suffering from cancer, infections or cardiovascular events 3. For example, prolonged intake of azathioprine has long been associated with increased incidence of tumors, particularly skin cancers in KTRs 4. At the same time, underimmunosuppression remains a clinical problem in that acute rejection is still frequent 5, albeit decreasing in magnitude, and chronic rejection is definitively significant and has a poor outcome 6. This means that there is an important clinical need to characterize biomarker signatures that could reliably identify patients who have developed a tolerant response to their graft. We and others have been trying to address this need 7, 8, 9, 10, 11.

The index patient group that is central to identifying tolerance is formed by those kidney transplant recipients (KTRs) who have challenged conventional clinical practice and have discontinued immunosuppressive medication while maintaining good graft function for years 12; these recipients are thus labeled as “tolerant.” In the process of biomarker discovery, these tolerant KTRs—free from IS for years—have always been compared with KTRs receiving various IS regimens, who are representatives of nontolerant patients. These differences in therapy between the groups imply that any previous signatures suffer from a systematic analysis flaw if the effects of the drugs have not been accounted for.

The final proof that any biomarker signature is indeed a signature of tolerance would only arise through a clinical trial whereby patients displaying the chosen signature would be weaned off IS and their graft would maintain good function. This definitive evidence for any signature tolerance is still missing in the literature. Consequently, the selection of possible signatures of tolerance need to abide by the most stringent quality requirements because testing them in prospective trials would put some patients at risk of late rejection or even at risk of irreversible graft damage if a misdiagnosis is made. To our knowledge, none of the referenced studies have yet attempted to directly address the fact that IS drugs affect gene expression despite available evidence 13, 14. Notably, a noninvasive signature of tolerance could also be used to evaluate the effectiveness of tolerance induction therapies that are currently under investigation in clinical trials 15, 16, 17.

Gene expression patterns found in the peripheral blood of KTRs can reflect at least two mechanisms: the response of the recipient's immune system to the presence of a highly immunogenic tissue (the transplanted graft) and the effect of IS treatment used to counteract the rejection process. Edemir et al 18 used a rodent model to describe gene expression patterns that support the spontaneous simultaneous activation of immune effector–related pathways and protective and immune counter‐regulatory mechanisms as a response to the allogeneic transplant. In humans, we and others have previously described a dysregulation of B cell–related genes in tolerant recipients—associated with the maintenance or expansion of transitional B cells in peripheral blood 8, 9—that elicited new avenues toward understanding the role of transitional B cells in tolerance 19, 20, 21. Differential expression profiles associated with IS treatment have been demonstrated by Erickson et al 22 in the context of transplantation in rats. Thus, when investigating gene expression markers of operational tolerance in humans, we need to ensure that we are isolating the natural counter‐regulatory immune mechanisms from those that reflect the IS drug intake, which could disappear after discontinuation of the drug.

We therefore undertook the current study to explore the effects of IS regimens on gene expression in peripheral blood and on our previously described signature of tolerance Sagoo et al 8 and hypothesized that adjusting for the confounding effects of IS drugs would provide more reliable biomarkers. We also hypothesized that by targeting immune responses via different mechanisms, IS drugs would have a differential effect on lymphocyte populations, particularly that of transitional B cells, which could bias the probability of tolerance estimates if differences in IS drug regimens are not accounted for. As a proof of concept of the effect of IS on lymphocyte subsets and gene expression, we have prospectively collected samples from patients who underwent steroid withdrawal owing to clinical reasons.

## Concise Materials and Methods

### Patients and samples

To collect the necessary evidence described in this article, we used samples from three patient cohorts:

- Cohort 1: We performed a reanalysis of the data from 71 European KTRs from the previously published Indices of Tolerance (IoT) study. This patient cohort had been used to discover the original biomarker signature and comprised 11 tolerant recipients, 51 stable patients (30 on standard triple therapy, 10 who had never received a CNI and 11 on low doses of prednisone), 9 patients with biopsy‐proven chronic rejection and 19 healthy controls; these patients are all thoroughly described in reference 8.
- Cohort 2: Cohort 2 is a novel observational case‐control cohort from the Genetic Analysis of Molecular Biomarkers of Immunological Tolerance (GAMBIT) study (Research Ethics Reference: 09/H0713/12). The cohort comprised tolerant (n = 14; detailed description in Table S3), stable (n = 190) and chronic rejection (n = 36) patients and healthy controls (n = 12). At least two blood samples—6 months apart on average—were obtained from each individual; these are identified in the text as time point 1 and time point 2.
- Cohort 3: Cohort 3 is a prospective cohort from the same GAMBIT study (Research Ethics Reference: 09/H0713/12) that included stable patients who, early posttransplant, were undergoing steroid withdrawal owing to clinical reasons. These patients were selected exclusively based on clinical criteria and were recruited from the London and Portsmouth hospitals. Patient selection and steroid withdrawal were conducted according to local clinical practice. Samples were collected before and 2 to 6 months after complete steroid withdrawal.

Patient characteristics from cohorts 2 and 3 are described in Table 1, and further clinical details are given in Data S1.

**Table 1:** Clinical and demographic characteristics of the patients from the GAMBIT study

Clinical parameters	Retrospective cohort					Prospective cohort
	Tolerant^1^	Stable^2^	Chronic^2^ rejector	Total retrospective	Healthy controls^2^	Steroid^3^ withdrawal
N	14	190	36	240	12	16
% female	21.4%nnn	32.6%	36.1%	32.5%	16.7%nnn	31.3%nnn
% deceased	50.0%nnn	66.5%	65.7%	65.4%	–	43.8%^nn^
Years posttransplant^4^	17.5* (2.2, 30.8)	12.5 (4.2, 36.7)	6.9 (1.3, 27.8)	11.8 (1.3, 36.8)	–	1.2*** (0.04, 24.6)
Age^5^	48.8nnn (15.2)	50.3 (13.4)	43.9 (13.6)	49.2 (13.7)	47.1nnn (14.9)	41.6^n^ (14.4)
eGFR^5^	57.9nnn (14.4)	64.1 (23.0)	32.4 (13.0)	59.2 (24.2)	–	64.9^nn^ (17.8)
Nr HLA‐MM^6^	3 (0–5)nnn	2 (0–6)	3 (0–6)	3 (0–6)	–	2^nn^ (0–4)
% DSA	21.4%	9.5%^nn^	44.4%^nn^	15.4%	–	0.0%
IS^7^^,^ 8						
% on Tac	–	26.8%	80.6%	35.4%	–	50.0%nnn
% on Cyc	–	46.3%	8.3%	40.3%	–	37.5%nnn
% on Aza	–	32.6%	13.9%	29.6%	–	0.0%
% on MMF	–	44.7%	63.9%	47.8%	–	93.7%***
% on Pred	–	41.6%	69.4%	46.0%	–	100.0%***
IS dose^8^						
Tac, ug/L	–	3.8 (2.1)	5.7 (4.1)	4.5 (3.1)	–	7.9^n^ (3.9)
Cyc, ug/L	–	157 (73)	158 (38)	157 (72)	–	208nnn (38)
Aza, mg/day	–	87 (36)	105 (45)	89 (37)	–	–
MMF, mg/day	–	1200 (450)	1112 (482)	1182 (457)	–	1483*** (522)
Pred, mg/day	–	4.8 (1.8)	6.7 (3.1)	5.3 (2.4)	–	6.1* (5.3)
WBC × 10^−9^ 5	7.3nnn (1.8)	7.2 (2.2)	7.1 (2.5)	7.2 (2.3)	–	9.6^n^ (4.7)
Lymph × 10^−9^ 5	1.8^n^ (0.6)	1.5 (0.6)	1.4 (0.9)	1.5 (0.7)	–	1.8nnn (1.1)
n with two samples	11	119	29	159	–	16

Healthy controls were age and gender matched to tolerants.

Aza, azathioprine oral dose; Cyc, cyclosporine A trough levels; DSA, Donor Specific Antibodies; eGFR, estimated Glomerular Filtration Rate; GAMBIT, Genetic Analysis of Molecular Biomarkers of Immunological Tolerance; IS, immunosuppression; Lymph, Lymphocytes; MM, mycophenolate mofetil; MMF, mycophenolate mofetil oral dose; Nr HLA‐MM, Number of Human Leucocyte Antigen mis‐matches (out of 6); Pred, prednisone oral dose; Tac, tacrolimus trough levels; WBC, White blood Cells.

^1^Statistical significance of the differences between tolerant patients and the combined group of stable and chronic rejectors.

^2^Statistical significance of the difference compared to tolerant patients.

^3^Statistical significance of the difference compared to the total retrospective cohort.

^4^Median (minimum, maximum).

^5^Mean (standard deviation [SD]).

^6^Median sum of HLA‐A, HLA‐B, and HLA‐DR mismatches (minimum and maximum).

^7^Percentage (%) from the total number of patients in the group (information on IS drugs was absent for eight stable patients [4.2%] and two chronic rejector patients [5.6%]).

^8^Mean (SD) doses at recruitment in mg are displayed.

The statistical significance of the difference between stable and chronic rejectors and the prospective steroid withdrawal cohort. ns > 0.05, *<0.05; ***<0.001; nonsignificant p‐values: ^n^0.05 to 0.1; ^nn^0.1 to 0.3; ^nnn^>0.3. ns, not statistically significant.

The characteristics of the patients in the prospective steroid withdrawal cohort 3 were largely comparable to the observational cohort 2 (Table 1), except that their time posttransplantation was significantly shorter (p < 0.001), none had Donor Specific Antibodies (DSA) and none were receiving azathioprine, whereas all but one were treated with mycophenolate mofetil (Table 1).

### RNA isolation, complementary DNA (cDNA) synthesis and reverse transcription quantitative real‐time polymerase chain reaction (RT‐qPCR)

Peripheral vein blood was drawn directly into Tempus Blood RNA Tubes (Life Technologies, Paisley, UK) and stored at −20°C. RNA isolation, cDNA synthesis, RT‐qPCR conditions and primers are described in Data S1.

### Fluidigm platform

The expression levels of a set of target genes and three endogenous reference genes were measured in 470 RNA samples on the Fluidigm BioMark quantitative real‐time PCR (qPCR) platform (South San Francisco, CA) with a preamplification step to validate the signature rederived from the IoT cohort. Further details are given in Data S1.

### Flow cytometry

Peripheral blood mononuclear cells (PBMCs) were isolated and frozen immediately at −80°C. After 24 h, cells were transferred into liquid nitrogen −170°C and kept until use. Antibody panels and acquisition details are described in Data S1.

### Statistical analyses

**Figure 1:**
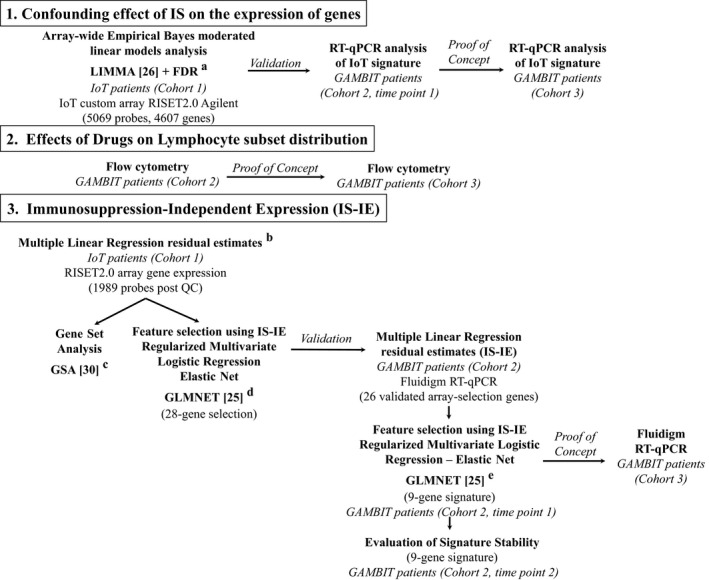
**Analysis strategy to demonstrate confounding effect of** **IS** **and how to avoid it.** A complete description can be found in Data S1. In summary: ^a^Probes with nonsignificant expression above background in more than 20% of the samples were filtered out. The effects of three factors were estimated: CNI: (ciclosporin [Cyc], tacrolimus [Tac], none), MMF/Aza (mycophenolate mofetil, azathioprine, none) and prednisone‐Pred‐(Pred; on steroids, off steroids). ^b^The model for the ith patient and the j_th_ Gene Set Analysis gene was y_ij_ = a_j_ + Cyc_i_ *β_Cycj_ + Tac_i_ *β_Tacj_ + Aza_i_ *β_Azaj_ + MMF_i_ *β_MMFj_ + Pred_i_ *β_Predj_ + ε_ij_. This was estimated using data from stable and chronic rejector patients. The resulting estimated residual ε_ij_ represents the IS‐independent gene expression (IS‐IE). The gene expression of tolerant patients and healthy controls was rescaled to IS‐IE by subtracting the intercept α_j_ from the raw expression. ^c^ GSA was used for the identification of differentially expressed biological pathways based on the curated list of gene sets from the Broad Institute (www.broadinstitute.org/gsea). Gene sets with an associated FDR below 10% were considered differentially expressed. ^d^In ElasticNet regression, a penalty is imposed on the regression coefficients, which is a combination of the penalties used in lasso and ridge regression. ElasticNet enables selection of genes (unlike ridge regression, which would preserve all genes) as well as gene groups irrespective of correlation (unlike lasso, which would select only one of a group of correlated genes). Model parameters were tuned using leave‐group‐out cross‐validation with a 65% training set and 100 resampling iterations, with the AUC as an accuracy measure, via the caret package in R 29. Prior to model estimation, missing values were imputed using K‐nearest neighbors. Genes for which the expression was not significantly above background (p > 0.01) in at least 80% of the samples were filtered out prior to analysis (3081 out of 5070 probes) to increase statistical power 10. ^e^Classification cutoffs were selected to ensure specificity above 0.85 while retaining sensitivity above 0.70. IS, immunosuppression; CNI, calcineurin inhibitors; Aza, azathioprine; MMF, mycophenolate mofetil; IS‐IE, IS‐independent expression; GLMNET, Lasso and Elastic‐Net Regularized Generalized Linear Models; GSA, Gene Set Analysis; FDR, false discovery rate; AUC, area under the receiver operating characteristic curve; GAMBIT, Genetic Analysis and Monitoring of Biomarkers of Immunological Tolerance; IoT, Indices of Tolerance; RT‐qPCR, reverse transcription quantitative real‐time polymerase chain reaction; RISET, Reprogramming the Immune System for the Establishment of Tolerance.

The analysis strategy is depicted in Figure 1. All statistical analyses (preprocessing of RT‐qPCR, Fluidigm and array data) were carried out in R software ( http://www.R-project.org/) 23, 24, 25. For individual gene expression, Ct values were normalized using DCt with respect to HPRT (hypoxanthine phosphoribosyltransferase) and were used as log2(2^−DCt^) values.

Associations of gene expression or of predicted probability of tolerance with IS drug intake were examined in linear regression models adjusting simultaneously for confounding by IS drugs other than the drug of interest. Bonferroni correction was applied for multiple comparisons between patients on and off different drugs.

### Elucidating the confounding effect of IS on gene expression

Details of the RISET 2.0 array and the preprocessing method have been published elsewhere 8. The array‐wide analysis of drug effects was carried out using empirical Bayes moderated linear models implemented in the Limma package 26. The Benjamini–Hochberg (BH) method was used for multiple testing correction. Genes for which expression was not significantly above background were filtered out prior to analysis to increase statistical power 27.

### Defining IS‐independent expression

The residuals of a linear regression model relating gene expression data from stable and chronic rejector patients to drug intake (binary yes/no) represent the IS‐independent expression (IS‐IE). The gene expression of tolerant patients and healthy controls was rescaled to IS‐IE by subtracting the intercept of that model from the raw expression.

### Defining the novel signature of tolerance

We used the regularized multivariate logistic regression method ElasticNet 28, 29 to select an optimal set of genes predictive of tolerance (as the number of genes was high relative to the number of patients), and considering many genes were correlated, thus invalidating the application of classical regression models. We compared the estimated IS‐IE in tolerant patients versus patients on IS (stable and with chronic rejection). In order to test the stability of the signatures, IS‐IE from the time point 2 samples was estimated based on the model trained on time point 1 samples, and classification accuracy was evaluated with the same cutoff.

For the identification of differentially expressed biological pathways, we carried out gene set analysis 30 using the curated list of gene sets from the Broad Institute (www.broadinstitute.org/gsea).

For the evaluation of predictive accuracy, an adjusted area under the receiver operating characteristic curve (AUC) was derived after fitting a linear regression model of the estimated probability of tolerance on drug regimen in IS patients and calculating the AUC for the residuals of this model 31.

Further details regarding the laboratory methods and statistical analysis are provided in Data S1.

## Results

### Immunosuppressants bias the expression of genes in peripheral blood

To study the effects of commonly used immunosuppressants on the expression of genes measured in peripheral blood, we first carried out a previously unattempted array‐wide analysis of drug effects using the expression measured on the original data from the IoT study 8. This revealed that 119 genes were differentially expressed in association with CNI drug intake and that 83 genes were associated with MMF and azathioprine intake, whereas only one gene was exclusively affected by steroid intake (Table S1).

To confirm the effect of IS, we then assessed the effect of IS on the expression of the previously published 10 genes of the signature using samples from stable patients on IS from cohort 2 (time point 1) of the GAMBIT study 8. A summary of the different IS regimens the patients were under is provided in Table S2. Prednisone and azathioprine showed statistically significant effects on the expression of 7 out of 10 of the previously described individual genetic markers of tolerance (Table 2). The effects of each drug were adjusted for the intake of other IS drugs.

**Table 2:** Effects of IS drugs on the published signature in stable patients from the GAMBIT study

Gene Symbols	Pred	Cyc	Tac	Aza	MMF
*PNOC*	0.11	0.10	0.04	0.76	1.00
*CD79b*	2.1 × 10^−04^	1.00	0.12	8.1 × 10^−04^	0.94
*TCL1A*	1.9 × 10^−06^	0.17	0.02	6.7 × 10^−16^	1.00
*H3ST1*	1.3 × 10^−04^	0.30	0.14	3.6 × 10^−05^	0.20
*SH2DB1*	0.42	1.00	1.00	<2.0 × 10^−16^	0.11
*TLR5*	4.0 × 10^−03^	1.00	0.09	1.00	1.00
*MS4A1*	3.0 × 10^−03^	0.73	0.18	1.1 × 10^−04^	1.00
*FCRL1*	1.7 × 10^−04^	1.00	0.73	1.1 × 10^−10^	1.00
*FCRL2*	5.7 × 10^−04^	1.00	0.15	1.6 × 10^−05^	1.00
*FoxP3/AMann*	0.69	0.16	9.0 × 10^−03^	1.00	1.00

p‐values for comparisons of stable patients on and off each drug are derived after adjustment in a linear regression model for all other drugs/drug groups. The p‐values for the Cyc/Tac group (subgroups No‐Cyc/Tac, Cyc, and Tac) and for the Aza/MMF group (subgroups No‐Aza/MMF, Aza, and MMF) were adjusted for multiple comparisons with Bonferroni correction.

Aza, azathioprine oral dose; Cyc, cyclosporine A trough levels; GAMBIT, Genetic Analysis of Molecular Biomarkers of Immunological Tolerance; IS, immunosuppression; MMF, mycophenolate mofetil oral dose; Pred, prednisone oral dose; Tac, tacrolimus trough levels.

To measure the magnitude of these effects, we calculated the percentage of variance in the expression of each gene explained by drug effects (R^2^ values from linear regression models based on data from the GAMBIT cohorts 2 and 3; Table S4). For 5 genes among the 10 in the original IoT signature, the percentage of expression explained by drugs was at or higher than 10% and up to 27% (*CD79b*,* TCL1A*,* SH2DB1*,* FCRL1*, and *MS4A1*), further strengthening the argument that the expression of genes included in the IoT signature was influenced by IS drug regimens.

**Figure 2:**
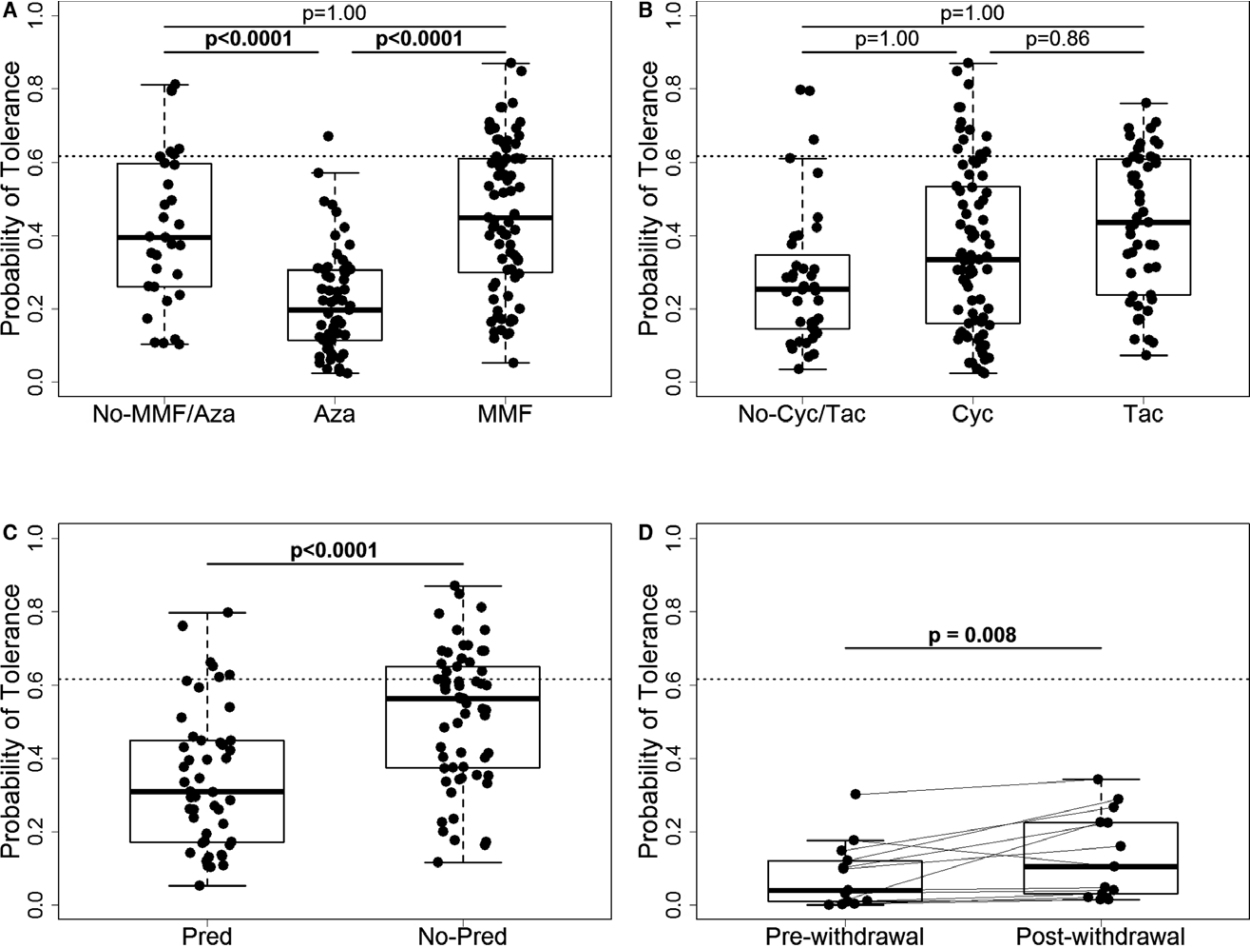
**Effects of immunosuppression (** **IS** **) drugs on the estimated probability of tolerance based on the 10‐gene algorithm from IoT (gene expression measured by RT‐qPCR in patients from the** **GAMBIT** **study, cohorts 2 and 3).** (A) Effect of antiproliferative drug intake in the stable patients group (n = 171) (No‐MMF/Aza patients not receiving MMF nor Aza n = 31), Aza n = 61, MMF n = 79. (B) Effect of CNI drug intake in the stable patients group (n = 171) (No‐Cyc/Tac patients not receiving Cyc nor Tac n = 40, Cyc n = 82, Tac n = 49). (C) Effect of prednisone intake on the estimated probability of tolerance in stable patients off azathioprine (n = 110) (Pred n = 48, No‐Pred n = 62). (D) Comparison of paired samples prewithdrawal and 3 to 6 months postwithdrawal completion from patients who have undergone clinically driven steroid withdrawal (n = 13 due to missing samples, none receiving azathioprine). The p‐values for each drug were derived after adjustment in a linear regression model for effects of all other drugs. The p‐values for CNI drugs and for antiproliferative drugs were adjusted for multiple comparisons with Bonferroni correction. The p‐values for comparisons pre– and post–steroid withdrawal were derived from a Wilcoxon matched pairs test. Cyc, ciclosporin; Tac, tacrolimus; Pred, prednisone/prednisolone; Aza, azathioprine; IS, immunosuppression; IoT, Indices of Tolerance study; RT‐qPCR, reverse transcription quantitative real‐time polymerase chain reaction; GAMBIT, Genetic Analysis of Molecular Biomarkers of Immunological Tolerance; MMF, Mycophenolate mofetil. Probability of tolerance cutoff was 0.62.

We then calculated the probability of tolerance for each patient based on the RT‐qPCR expression in peripheral blood samples. We used the gene expression from the previously published IoT signature, as described 8 for patients from cohort 2, time point 1, of the GAMBIT study. We observed that this probability was undeniably and significantly associated with the drug exposure of the patients (Figure 2). The probability was significantly lower in stable patients treated with azathioprine compared to patients off antiproliferative (p < 0.0001) and patients on MMF (p < 0.0001) (Figure 2A). This probability was not influenced by the intake of CNIs once the effects of prednisone and azathioprine were accounted for (Figure 2B). Similar to azathioprine, the administration of prednisone was associated with a significantly lower estimated probability of tolerance (p < 0.0001) in stable patients in GAMBIT cohort 2 (Figure 2C; note that the patients shown in this plot are all off azathioprine).

To further confirm this observation, we used the gene expression from samples in the prospective GAMBIT cohort 3 (p = 0.008). Indeed, the estimated probability of tolerance significantly increased after steroid withdrawal (Figure 2D; please note that no patient in this group was receiving azathioprine).

The AUC for the probability of tolerance estimates from the comparison between tolerant and IS‐treated patients from GAMBIT cohort 2, based on the IoT signature, was 0.89 (95% confidence interval [CI]: 0.83–0.94). When the AUC was adjusted for the effects of IS drugs, it became significantly lower: 0.77 (95% CI 0.67–0.86; p = 0.032). This provided further evidence for a confounding effect of IS regimens in the expression of these genes.

Therefore, we have demonstrated, in three completely independent cohorts (1, 2 and 3), evidence of drug confounding or bias in the gene expression of our previously identified tolerance signature.

### Immunosuppressants affect the transitional B cell subset

**Figure 3:**
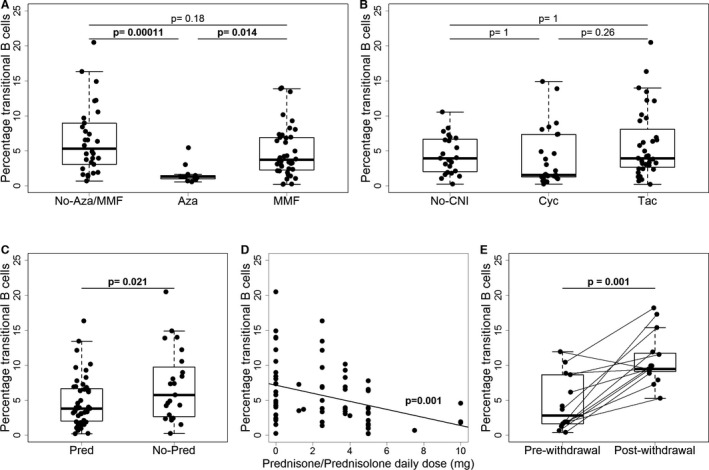
**Percentage of** **CD** **24hi** **CD** **38hi (transitional B cells) within the live** **CD** **20 + CD19 + B lymphocytes and** **CD** **27‐IgD+IgM+ gate in peripheral blood of patients from the** **GAMBIT** **study, cohorts 2 and 3.** (A) Effect of antiproliferative drug intake on transitional B cell subset size in the stable patients group (n = 111) (No‐MMF/Aza n = 33, Aza n = 24, MMF n = 54). (B) Effect of CNI drug intake (n = 111) (No‐Cyc/Tac n = 28, Cyc n = 41, Tac n = 42). (C) Effect of prednisone intake on the transitional B cell subset size in stable patients off azathioprine (n = 87) (Pred n = 53, No‐Pred n = 34). (D) Effect of prednisone total daily dose (mg) in stable patients off azathioprine. (E) Comparison of paired samples prewithdrawal and 3 to 6 months postwithdrawal completion from patients who have undergone clinically driven steroid withdrawal (n = 16, none receiving azathioprine). The p‐values for each drug are derived after adjustment in a linear regression model for all other drugs/drug groups. The p‐values for CNI drugs and for antiproliferative drugs were adjusted for multiple comparisons with Bonferroni correction. The p‐values for comparisons pre– and post–steroid withdrawal were derived from a Wilcoxon matched pairs test. Cyc, ciclosporin; Tac, tacrolimus; Pred, prednisone/prednisolone; Aza, azathioprine; IS, immunosuppression; IoT, Indices of Tolerance study; GAMBIT, Genetic Analysis of Molecular Biomarkers of Immunological Tolerance; MMF, Mycophenolate Mofetil; CNI, Calceneurin Inhibitors.

Whole blood gene expression data are greatly influenced by the repertoire of circulating lymphocyte subsets. To determine whether the previously described association of tolerance with a relative increase in circulating transitional B cells in tolerant recipients might also be confounded by IS, we performed flow cytometry analysis of peripheral blood cells from the patients included in GAMBIT cohort 2 (for gating, see Figure S2). The effect of IS drugs was clearly evident in the percentage of transitional B cells within the naive B cell population (Figure 3), and this effect was similarly observed when measuring absolute numbers of cells in a subset of the recipients (Figure S3). Notably, the pattern of the changes closely resembled the effect of IS drugs on the estimated probability of tolerance based on the IoT signature. Stable patients on azathioprine (Figure 3A) and those on prednisone (Figure 3C) showed lower percentages of transitional B cells than patients off each of these drugs, whereas CNI drugs showed no effect (Figure 3B). The effect of prednisone showed a clear dose–response relationship (Figure 3D), and this was confirmed in the prospective steroid withdrawal GAMBIT cohort 3 (Figure 3E). The percentage of total B cells in periphery significantly decreased only for those patients taking azathioprine (data not shown). Changes in the opposite direction were observed in the percentages of T cells from lymphocytes. These were increased in stable patients taking azathioprine or prednisone but were unaffected by CNI drugs (Figures S1A, B and D). However, the evidence for a dose–response effect of prednisone was very weak, and there were no differences in the percentages of peripheral blood T cells in the prospective steroid withdrawal cohort 3 (Figures S1E and F).

### Development of a novel immunosuppression‐independent gene expression signature of tolerance

Having demonstrated that the IS drug regimen is a confounder for the association between gene expression levels and the predicted probability of tolerance, we concluded that for a predictive test of tolerance to be clinically applicable and unbiased, IS effects on gene expression must be accounted for in the predictive algorithm. We used the array data from the IoT study (cohort 1) as a “discovery” set. We obtained the residuals of a multivariate linear regression model for IS drugs per each gene in the array. We used this to estimate the IS‐independent gene expression (IS‐IE) per gene.

**Figure 4:**
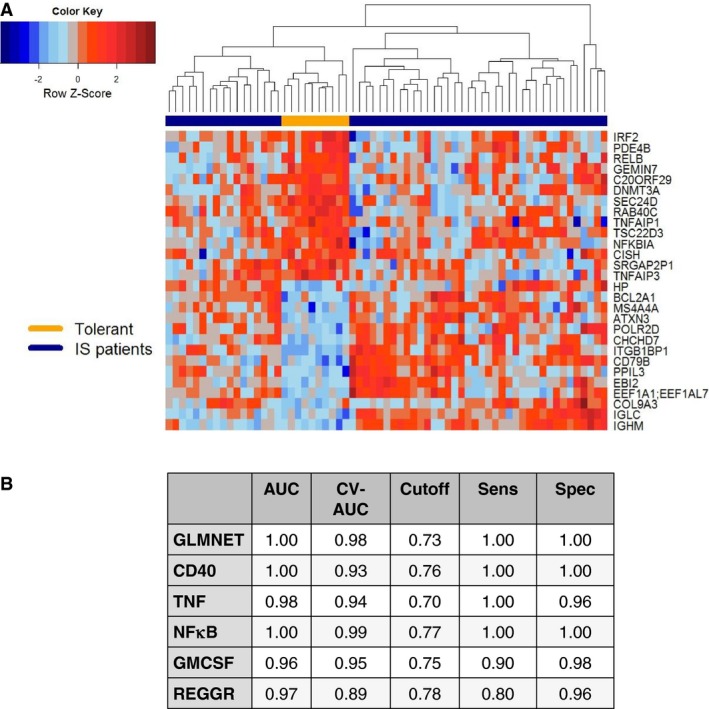
**Discovery of a new set of** **IS** **‐independent markers of tolerance.** (A) Heat map showing patterns of residual gene expression from 28 genes selected using ElasticNet on array data from the IoT cohort, comparing tolerant versus immunosuppressed recipients. (B) Predictive accuracy of multivariate predictive sets of genes. GLMNET refers to the 28‐gene set discovered via ElasticNet analysis. The remaining sets were selected from those molecular pathways shown to be differentially expressed via gene set analysis. AUC, area under the receiver operating characteristic curve; CV‐AUC, cross‐validated AUC; IoT, Indices of Tolerance; Sens, sensitivity; Spec, specificity; GLMNET, Elastic Net Regularized General Linear Model; REGGR, Glucocorticoid receptor regulatory network; TNF, Tumor Necrosis Factor Pathway; CD40, CD40L Signaling Pathway; NFKB, NF‐kappaB Signaling Pathway; GMCSF, GMCSF‐ mediated signaling events.

We used the estimated IS‐IE to select an optimal set of genes predictive of tolerance. To enable applicability of the signature in a clinical setting, we restricted the selection to a maximum of 30 genes. The resulting set of 28 IS‐independent genes provided excellent predictive accuracy (Figures 4A and B; AUC, sensitivity and specificity of 1; cross‐validated AUC = 0.98).

Subsequently, using this IS‐IE expression, gene set analysis revealed five differentially expressed biological pathways: nuclear factor kappa B (NFκB), CD40, tumor necrosis factor (TNF), Granulocyte‐macrophage colony‐stimulating factor (GMCSF), and Glucocorticoid receptor regulatory network (REGGR; of which only CD40 and NFκB could be identified as preferential gene pathways in B cells). The resulting prediction sets also provided excellent predictive accuracy to identify tolerant recipients (Figure 4B).

### Validation of the new signature on Fluidigm platform in samples from the GAMBIT study

We chose to validate and further refine the new IS‐IE gene set using the Fluidigm platform, an RT‐qPCR–based assay, in samples from patients in GAMBIT cohort 2 (time point 1). Quality control criteria were met by 26 genes.

A set of 9 genes out of the 26 was selected by ElasticNet as optimal to predict tolerance (see Data S1). Table 3 shows the validated IS‐IE nine‐gene list. Please note that drugs explained very little of the variance of their expression (see R^2^ in Table S5). No gene overlapped with the previous IoT signature.

**Table 3:** Immunosuppression‐independent gene signature

Symbol	Gene name	Molecular function	Biological processes	Documented protein expression in
ATXN3 ↓	Ataxin 3	Ubiquitin‐specific protease activity	Protein metabolism	Caudate nucleus, cerebellum frontal cortex, pons, ubiquitous
BCL2A1 ↓	BCL2‐related protein A1	Receptor signaling complex scaffold activity	Apoptosis	B cell 49, bone marrow, colon, intestine, leucocyte, lymph node, ovary, spleen, T cell
EEF1A1 ↓	Eukaryotic translation elongation factor 1 alpha 1	Transcription regulator activity	Regulation of cell cycle	B cell 50, islets of Langerhans, lachrymal gland, leukocyte, monocyte, neutrophil, plasma, saliva, semen, skeletal muscle, tear
GEMIN7 ↑	Gem (nuclear organelle) associated protein 7	Ribonucleoprotein	Regulation of nucleobase, nucleoside, nucleotide and nucleic acid metabolism	Spinal cord tissues
IGLC1 ↑	Immunoglobulin lambda constant 1 (Mcg marker)	Antigen binding	Immune response	B lymphocytes 51
MS4A4A ↑	Membrane‐spanning 4‐domains, subfamily A, member 4A	–	–	Intestine and colon
NFκBIA ↓	Nuclear factor of kappa light polypeptide gene enhancer in B cells inhibitor, alpha	Transcription regulator activity	Regulation of nucleobase, nucleoside, nucleotide and nucleic acid metabolism	Neutrophil, T cell
RAB40C ↑	RAB40C, member of RAS oncogene family	GTPase activity	Cell communication, signal transduction	Platelets, liver, heart, kidney, plasma
TNFAIP3 ↓	Tumor necrosis factor, alpha‐induced protein 3 (A20, Zin finger protein A20)	Transcription regulator activity	Regulation of nucleobase, nucleoside, nucleotide and nucleic acid metabolism	Macrophages

↓, IS‐free gene expression downregulated in tolerant patients compared to patients on IS; ↑, IS‐free gene expression upregulated in tolerant patients compared to patients on IS.

**Figure 5:**
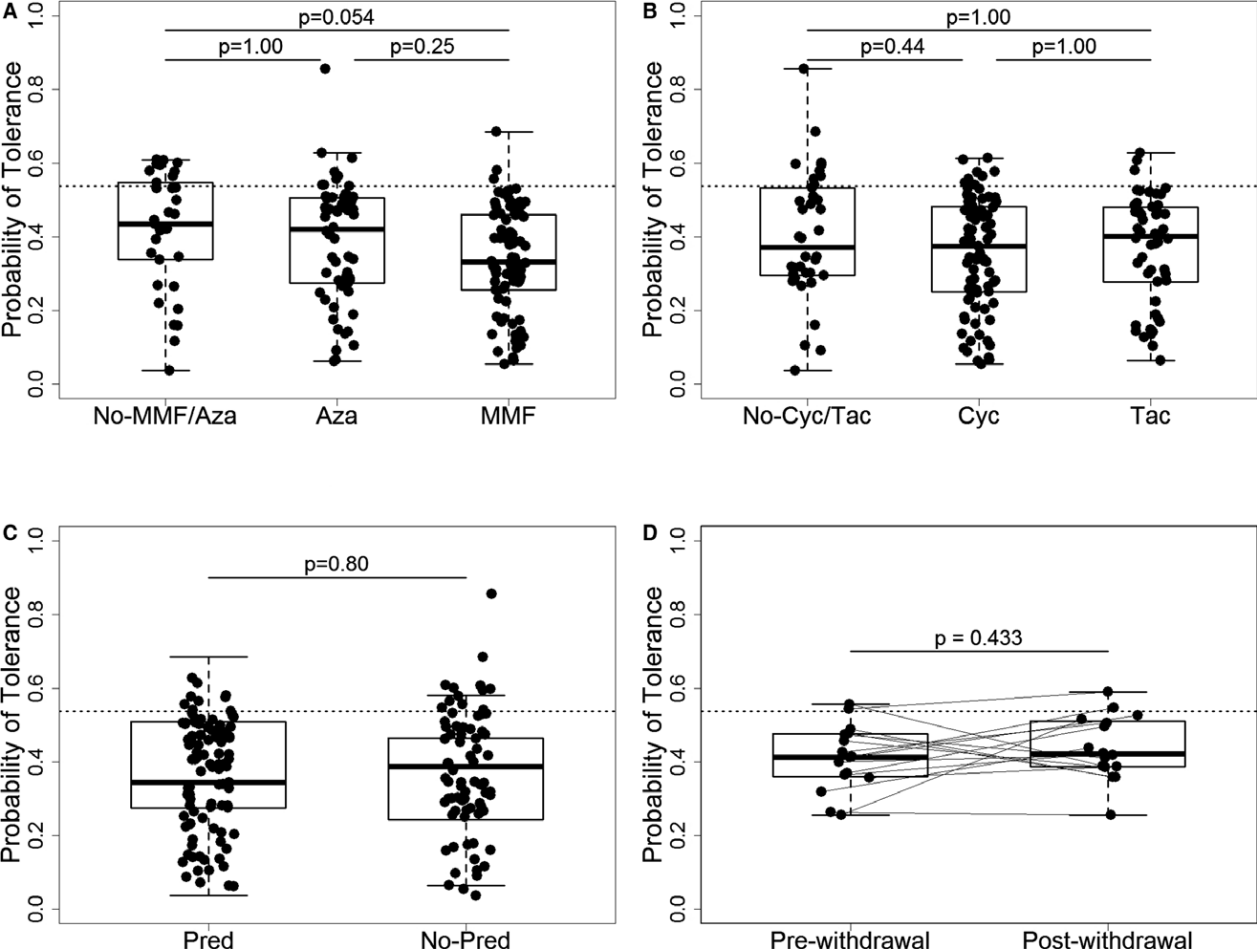
**The estimated probability of tolerance, based on the new nine‐gene algorithm, is independent of immunosuppressive (IS) drugs (gene expression measured in Fluidigm platform in patients from the** **GAMBIT** **study, cohorts 2 and 3).** (A) Effect of antiproliferative drug intake on estimated probability of tolerance in stable patients (n = 173) (No‐MMF/Aza n = 33, Aza n = 58, MMF n = 82). (B) Effect of CNI drug intake on estimated probability of tolerance in stable patients (n = 173) (No‐Cyc/Tac n = 38, Cyc n = 85, Tac n = 50). (C) Effect of prednisone intake on estimated probability of tolerance in stable patients off azathioprine (n = 119); (n = 115) (Pred n = 52, No‐Pred n = 63). (D) Comparison of estimated probability of tolerance, in paired samples prewithdrawal and 3 to 6 months postwithdrawal completion from patients who have undergone clinically driven steroid withdrawal (n = 16, none receiving azathioprine). The p‐values for each statistical comparison were derived after adjustment in a linear regression model for all other drugs/drug groups. The p‐values for CNI drugs and for antiproliferative drugs were adjusted for multiple comparisons with Bonferroni correction. The p‐values for comparisons pre– and post–steroid withdrawal were derived from a Wilcoxon matched pairs test. Cyc, ciclosporin; Tac, tacrolimus; Pred, prednisone/prednisolone; Aza, azathioprine; IS, immunosuppression; IoT, Indices of Tolerance study; GAMBIT, Genetic Analysis of Molecular Biomarkers of Immunological Tolerance; MMF, Mycophenolate Mofetil; CNI, Calcineurin Inhibitors. Probability of tolerance cutoff was 0.54.

The new IS‐IE estimated probability of tolerance was independent of IS regimen—and therefore unconfounded—in stable patients (p > 0.05 for all drugs) from GAMBIT cohort 2 (Figures 5A, B and C). Further, in the prospective GAMBIT cohort 3, we could demonstrate that steroid withdrawal does not affect the expression of this new gene signature (Figure 5D).

The predictive accuracy of the new signature, as evaluated by the AUC, was 0.93 (95% CI: 0.86–1.0) and 0.81 after optimism correction via cross‐validation. A classification cutoff of 0.54 was selected to maximize specificity for patient safety (sensitivity of 0.92, specificity of 0.88).

Based on the IS‐IE signature, 20 out of 173 patients from cohort 2 on IS with stable function were identified as “probably tolerant” (11.6%). These patients were different individuals from those identified with the IoT signature (n = 25) (only two overlapping). Comparison of the two groups (Tables S6 and S7; Data S1) revealed, importantly, that the IS‐IE selection had significantly longer time posttransplantation, making them more comparable to the index group of tolerant patients.

Evaluation of the stability of the signature developed in cohort 1 (by estimating its predictive accuracy in two time points in cohort 2) showed satisfactory performance (AUC time point 2 = 0.83; 95% CI: 0.67–0.99). Additionally, a nonsignificant McNemar's test indicated that binary classification is stable across repeated samples (p = 0.095), meaning that classifications at one time point are not significantly different from those at a subsequent time point. Furthermore, the continuous predicted probability of tolerance does not change significantly between time points (Figure S4).

The genes included in the new signature participate in cellular pathways such as regulation of nucleic acid metabolism (GEMINI7, NFκB1A, TNFIP3), cell communication activities (RAB40C) and transcription factor activity (EEF1A1, NFκB1A, TNFIP3). Still, the protein expression derived from three of the genes has been observed in B cells and in other cells (BCL2A1, EEF1A1) or in B cells exclusively (IGLC1; see Table 3).

### The new IS‐independent signature is differentially expressed in healthy controls

**Figure 6:**
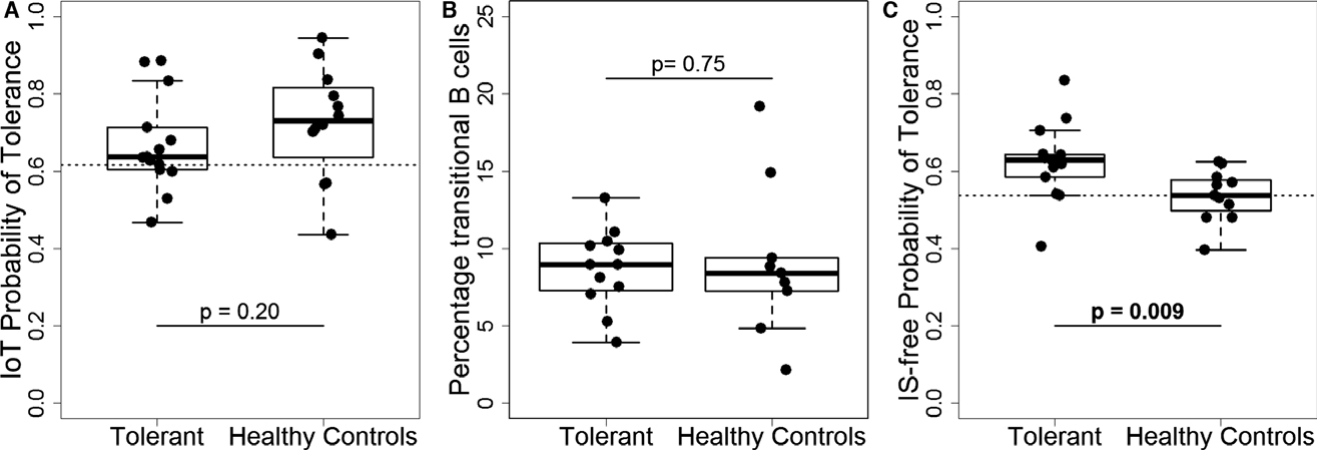
**Comparison of gene expression signatures between tolerant recipients and healthy controls in patients from the** **GAMBIT** **study (cohort 2).** (A) Estimated probability of tolerance based on the 10‐gene algorithm from IoT (gene expression measured by RT‐qPCR, cutoff of 0.62). (B) Percentage of CD24hiCD38hi (transitional B cells) within the live CD20 + CD19 + B lymphocytes and CD27‐IgD+IgM+ gate in peripheral blood. (C) Estimated probability of tolerance based on the new nine‐gene algorithm (gene expression measured in Fluidigm platform, cutoff of 0.54). Tolerant recipients (n = 14 for A and B, n = 13 for C); healthy controls (n = 12 for A and B, n = 11 for C). The p‐values were derived from a Wilcoxon test for comparison of independent samples. GAMBIT, Genetic Analysis of Molecular Biomarkers of Immunological Tolerance; RT‐qPCR, reverse transcription quantitative real‐time polymerase chain reaction; IoT, Indices of Tolerance.

An important limitation of the previously described signatures is their inability to differentiate between tolerant recipients and healthy controls (as illustrated in Figure 6A for the IoT signature). Similarly, we have observed no difference in the transitional B cell percentages in peripheral blood between these two groups (Figure 6B). Importantly, the predicted probability of tolerance based on the new signature was higher in tolerant patients compared to healthy controls (Figure 6C). This suggests that the method proposed herein and the resulting novel signature indeed capture an underlying predisposition to tolerance rather than the absence of IS drugs. Comparisons of the predicted probabilities of tolerance in tolerant patients and healthy controls with the corresponding cutoffs for the IoT and the IS‐IE signatures are shown in Table S8 and are discussed in Data S1.

## Discussion

A number of studies, including our own, have suggested gene expression signatures of tolerance in kidney transplantation 7, 8, 9, 11, 32 or have described differential expression of smaller gene sets 11, 33, 34. None had assessed the confounding effect of IS. Baron et al 10 recently completed a comprehensive review of all public data and concluded that the expression of a set of 20 genes—mostly expressed by B cells—may be used as a standardized tool for personalized medicine in KTRs. Four genes from our IoT signature (*TCL1A*,* MS4A1*,* FCRL2*, and *CD79B*) were included in this 20‐gene set, but we now demonstrate that their expression is highly affected by azathioprine and prednisone. Similarly, it has been shown, in an independent cohort, that azathioprine affects the three‐gene signature proposed by the Immune Tolerance Network 35. We have shown in our results, using the published signature of tolerance, that KTRs who were maintained off steroids, on MMF and on tacrolimus were being identified more frequently as possible tolerant recipients.

Therefore, we asked, “Was this tolerance or response to immunosuppressive therapy?” We have now demonstrated that it is highly likely that it was the latter. Consequently, we have developed and validated a new noninvasive gene expression signature of tolerance that is independent of IS drug effects and that additionally differentiates tolerant patients from healthy controls. This dictates that further analysis of tolerance signatures using the correction proposed herein (or a similar method) needs to be considered.

Additionally, we have demonstrated that the percentages of transitional B cells in peripheral blood, which had been described as characteristic of tolerant recipients, were also significantly affected by IS drugs. We have not addressed the specific mechanism by which each immunosuppressant affects the intracellular pathways in individual lymphocyte subsets, as this would require longer‐term studies and is beyond the scope of the current one. However, commonly used immunosuppressants, such as CNIs, which are fundamentally aimed at inhibiting T cell activation and have only an indirect effect on B cell activation, consistently showed little effect on gene expression, the percentage of transitional B cell subset size or the estimated probability of tolerance in our study after adjustment for intake of other IS drugs. When specifically addressed, transitional B cells exhibit the capacity to decrease anti‐inflammatory responses and produce anti‐inflammatory cytokines 21, 36. Therefore, we do not question the important functionality that transitional B cells may play in transplantation tolerance, but we believe that the evidence of the role of B cells in tolerance needs further scrutiny, particularly regarding the immunosuppression effects.

While there is no clear major genetic pathway connecting the genes included in the new IS‐independent signature, literature reports suggest that at least some of these genes have a mechanistic relevance to tolerance. For example, a polymorphism in the NFκBIA gene resulting in upregulation has been associated with higher rates of acute liver transplant rejection 37, and BCL2A1 has been shown to be a transcriptional target for NFκB 38. In our study, both genes were downregulated in tolerant patients. IGLC1 has been included in the expansion of the B cell signature of tolerance in KTRs 39. Upregulation of MS4A4A and RAB40C (upregulated in tolerant patients in our study) has been associated with macrophage activation 40, 41. TNFAIP3 (A20) is an NFκB regulatory protein and its expression has been associated with outcome prediction in kidney transplantation 42, but the regulation of its expression and function in inflammatory responses has been shown to be complex 43, 44. The association found herein of the downregulation of this gene with operational tolerance merits further investigation.

External validation of the presented signature in other independent cohorts would strengthen the confidence in the generalizability of the results and would allow final calibration before translation into clinical practice. Such studies would require the collection of detailed clinical phenotype data in parallel to the gene expression data (best approached in a prospective manner). We are part of two European consortia that will provide data for this further validation 15, 45, 46.

In conclusion, this study emphasizes the importance of assessing and correcting for the effect of diverse IS regimens on gene expression–based biomarker signatures. Using this correction, we identify a novel—and, arguably, a more clinically robust—signature of operational tolerance, which we have validated in independent and extensive cohorts of KTRs. Moreover, in our prospective validation cohort, the estimated probability of tolerance remained unchanged after steroid withdrawal, supporting the view that the new signature highlights natural counter‐regulatory mechanisms and excludes transient alterations of the immune effector pathways by IS drugs. Further evidence that our approach is uncovering tolerance‐related responses is the fact that the estimated probability of tolerance in tolerant patients is higher than that of healthy controls, in agreement with studies demonstrating the involvement of an active immune response in tolerance 47.

We are aware that these results do bring into question previous published evidence, and we have demonstrated the effect of IS drugs on our own published signature. Transplant physicians will require confidence in any novel clinical‐grade biomarker set of kidney transplantation tolerance, such as the new one described in this article, prior to embarking on clinical trials of IS weaning or minimization. Such trials are critical if we aim to reduce cancer risk and increase long‐term survival with improved quality of life for KTRs. In the current climate of stratified medicine, these findings may also be relevant to autoimmune diseases and other disorders in which IS is a prevalent drug therapy.

## Author Contributions

I.R.‐M. performed all the statistical analysis, wrote the main part of the manuscript and contributed to the acquisition of funds. E.N.‐L. worked specifically on the B cell flow cytometry studies and contributed to the review of the manuscript. P.M., M.R., S.N., Y.K., and N.S. significantly contributed to the experiments and experiment planning. S.C. contributed to statistical analysis and the writing of the manuscript. R.H., S.B., R.B., D.B., S.C., D.G., S.G., R.L., P.K., P.B.M., S.M., I.M., W.M., M.M., R.P., S.P.K., D.S., M.D.S., B.T., and O.V. contributed the clinical and follow‐up data of the patients and contributed to the review of the manuscript. R.I.L. and G.M.L. partially contributed to the funding provisions, mentoring, project overview and review of the manuscript. M.P.H.F. designed the study, obtained funding, managed the team, and contributed to and supervised the writing of the manuscript.

## Disclosure

The authors of this manuscript have no conflicts of interest to disclose as described by the *American Journal of Transplantation*.

## Supporting information


**Data S1:** Materials and methods.
**Table S1:** Genes whose expression is affected by immunosuppressive drugs (Excel file attached at the end).
**Table S2:** Distribution of drug regimen in stable patients.
**Table S3:** Detailed clinical description of GAMBIT tolerant patients. GAMBIT, Genetic Analysis of Molecular Biomarkers of Immunological Tolerance.
**Table S4:** Assay list used for the RT‐qPCR test of the Indices of Tolerance gene list. RT‐qPCR, reverse transcription quantitative real‐time polymerase chain reaction.
**Table S5:** Gene list (reliable signal) with assay description used in the Fluidigm platform for ElasticNet selection of the immunosuppression‐free signature.
**Table S6:** Clinical and demographic characteristics of stable patients from the GAMBIT study classified as tolerant with the IoT and IS‐IE signatures (classification match only in two patients). GAMBIT, Genetic Analysis of Molecular Biomarkers of Immunological Tolerance; IoT, Indices of Tolerance; IS‐IE, Immunosuppression‐independent expression.
**Table S7:** Effects of IS drugs on predicted probability of tolerance according to the IoT and IS‐IE signatures in stable patients from the GAMBIT study. IS, Immunosuppression; IoT, Indices of Tolerance; IS‐IE, Immunosuppression independent gene expression: GAMBIT, Genetic Analysis of Molecular Biomarkers of Immunological Tolerance .
**Table S8:** Predicted probability of tolerance in tolerant patients and healthy controls in relation to the cutoff.
**Figure S1: Percentage of T cells (CD3+ cells within the live lymphocyte gate) in peripheral blood of patients from the GAMBIT cohort.** GAMBIT, Genetic Analysis of Molecular Biomarkers of Immunological Tolerance .
**Figure S2: Gating strategy for transitional B cells in the flow cytometer.**
**Figure S3: Absolute number of CD24hiCD38hi (transitional B cells) within the live CD20+ CD19+ B lymphocytes and CD27‐IgD+IgM+ gate in peripheral blood of patients from the GAMBIT study, cohort 2.** GAMBIT, Genetic Analysis of Molecular Biomarkers of Immunological Tolerance.
**Figure S4: Estimated probability of tolerance is stable over time.**
