# Supplementary material for: Biomarkers of Tolerance in Kidney Transplantation: Are We Predicting Tolerance or Response to Immunosuppressive Treatment?
Source: Am J Transplant. 2016 Aug 8;16(12):3443–57. doi: 10.1111/ajt.13932 (PMC5132071; doi:10.1111/ajt.13932)
Supplement: Supplementary file 1 — Data S1: Materials and methods. Table S1: Genes whose expression is affected by immunosuppressive drugs (Excel file attached at the end). Table S2: Distribution of drug regimen in stable patients. Table S3: Detailed clinical description of GAMBIT tolerant patients. GAMBIT, Genetic Analysis of Molecular Biomarkers of Immunological Tolerance. Table S4: Assay list used for the RT‐qPCR test of the Indices of Tolerance gene list. RT‐qPCR, reverse transcription quantitative real‐time polymerase chain reaction. Table S5: Gene list (reliable signal) with assay description used in the Fluidigm platform for ElasticNet selection of the immunosuppression‐free signature. Table S6: Clinical and demographic characteristics of stable patients from the GAMBIT study classified as tolerant with the IoT and IS‐IE signatures (classification match only in two patients). GAMBIT, Genetic Analysis of Molecular Biomarkers of Immunological Tolerance; IoT, Indices of Tolerance; IS‐IE, Immunosuppression‐independent expression. Table S7: Effects of IS drugs on predicted probability of tolerance according to the IoT and IS‐IE signatures in stable patients from the GAMBIT study. IS, Immunosuppression; IoT, Indices of Tolerance; IS‐IE, Immunosuppression independent gene expression: GAMBIT, Genetic Analysis of Molecular Biomarkers of Immunological Tolerance . Table S8: Predicted probability of tolerance in tolerant patients and healthy controls in relation to the cutoff. Figure S1: Percentage of T cells (CD3+ cells within the live lymphocyte gate) in peripheral blood of patients from the GAMBIT cohort. GAMBIT, Genetic Analysis of Molecular Biomarkers of Immunological Tolerance . Figure S2: Gating strategy for transitional B cells in the flow cytometer. Figure S3: Absolute number of CD24hiCD38hi (transitional B cells) within the live CD20+ CD19+ B lymphocytes and CD27‐IgD+IgM+ gate in peripheral blood of patients from the GAMBIT study, cohort 2. GAMBIT, Genetic Analysis of Molecular Biomarkers of Imm [file AJT-16-3443-s001.docx]

Supplementary Materials:

- Supplementary Materials and Methods
- Supplementary Results
  - *Selection of the 9-gene signature*
  - *Important features of the new signature. Different patients are chosen with the new IS-independent signature.*
  - *Important features of the new signature. Studying the effect of drugs and time since transplantation.*
  - *Important features of the new signature. Estimated probabilities of tolerance in healthy controls and tolerant patients compared to the cut-off*
  - *Donor Specific Antibodies from Tolerant recipients*
- Supplementary Table 1: Genes whose expression is affected by immunosuppressive drugs (excel file attached at the end)
- Supplementary Table 2: Distribution of drug regimen in stable patients
- Supplementary Table 3: Detailed clinical description of GAMBIT tolerant patients
- Supplementary Table 4: Assay list used for the RT-PCR test of the Indices of Tolerance gene-list
- Supplementary Table 5: Gene-list (reliable signal) with assay description used in the Fluidigm platform for elastic net selection of the immunosuppression-free signature.
- Supplementary Table 6: Clinical and demographic characteristics of stable patients from the GAMBIT study classified as tolerant with the IoT and the IS-IE signatures (classification match only in two patients).
- Supplementary Table 7: Effects of IS drugs on predicted probability of tolerance according to the IoT and IS-IE signatures in stable patients from the GAMBIT study
- Supplementary Table 8: Predicted probability of tolerance in Tolerant patients and Healthy control in relation to the cut-off
- Supplementary Fig. 1. Percentage of T cells (CD3+ cells within the live lymphocyte gate) in peripheral blood of patients from the GAMBIT cohort.
- Supplementary Figure 2. Gating strategy for Transitional B cells in the flow cytometer.
- Supplementary Figure 3. Absolute number of CD24hiCD38hi (transitional B-cells) within the live CD20+CD19+ B lymphocytes and CD27-IgD+IgM+ gate in peripheral blood of patients from the GAMBIT study, cohort 2.
- Supplementary Figure 4. Estimated probability of tolerance is stable over time.

**Supplementary Materials and Methods**

*Patients and Study Design*

**Cohort 1**: Patients from the Indices of Tolerance study, (UK REC: 2002 / 6378) a retrospective case-control study, were all patients groups have been previously described (1).

**Cohort 2:** *Retrospective Case-Control* collection: The selected patient groups were as follows (Table 1 Main Section): *Tolerant (n=14)*: all previously-unidentified patients that have been completely off IS for longer than 12 months with <10% CRT rise since baseline. Individual Clinical details are given in Supplementary Table 3. Four tolerant patients had none out of six possible HLA mismatches (MM) in A, B and DR; none had 2 MM in A, only one had 2 MM in DR, the rest varied; while for one patient the donor HLA information was unknown. S*table (n=190)*: adult KTRs, with stable graft function (<15% change in mean eGFR in the last 12 months), that have been transplanted for longer than 4 years and that were maintained on any IS therapy (therapy regimens described in Supplementary Table1). *Chronic Rejector*s *(n=36)*: adult KTRs, more than 1 year post- transplant with increasing dysfunction that had undergone a graft biopsy in the previous 12 months and had been classified as having immunologically-driven chronic allograft nephropathy (Banff 2007 or higher). A minimum of two blood samples over 4 months apart were taken (median 6. 1 months, range 1. 0 – 27. 1 months). The IS regimen (or absence of) found in these patients at recruitment are the result of clinical decisions over the years in the 15 centers included in the study, or personal preferences in the case of some tolerant recipients. Different induction therapies may have been used and changes of medication were based on the respective local protocols and clinical findings. None of the patients had received a tolerance-induction strategy based on cell infusion or similar.

Patients in this cohort were predominantly males (68%), with 65% of deceased donors, and a median of 11. 8 years post-transplant. Tolerant patients (n=14, individually described in Supplementary Table2) had been transplanted for a median of 17. 5 years (marginally longer than chronic rejectors and stable patients (p=0. 034)) and had been IS-free for 1 to 22 years. None had been included in any of the previous published studies.

**Cohort 3:** *Prospective collection*: adult KTRs that were selected for programmed steroid withdrawal for clinical reasons (either standard protocol of their center attempted at 1 year after transplant or due to kidney-pancreas simultaneous transplantation, diabetes or overweight or subject to the clinical criteria of the clinician in charge). The standard protocol involved 1 mg dose decrease per month. For this group at least two samples were obtained, the first whilst the patient was on steroids (min 3 mg/day) and the second 2 – 6 months after steroid cessation.

In the GAMBIT Study (REC: 09/H0713/12) sample collection all patient recruitment and sample collection started in September 2009 and finished in December 2014. All samples were taken when patients did not have signs or symptoms of active infectious disease and their most recent peripheral blood hemoglobin levels were > 10 mg/dL. Patients were excluded if they had been diagnosed or treated for cancer in the previous 5 years prior to sampling. All patients gave their consent to the study protocol and signed this.

All laboratory data was generated at the MRC canter for Transplantation. All clinical data was collected by consulting electronic and paper records from the centers were the patients were being followed up. Research nurses and clinicians in charge of the centers involved provided the data to the coordinating center (KCL).

*Flow Cytometry*

Peripheral blood samples were collected in EDTA-coated Vacutainers (BD) and samples were processed in all cases within 24 hours of venesection. Peripheral blood mononuclear cells (PBMCs) were obtained by density gradient centrifugation using Lymphocyte Separation Medium (PAA Laboratories). Cells were washed and resuspended in 10% DMSO (Sigma-Aldrich) and human serum (Biosera) and frozen immediately at –80°C. After 24 hours cells were transferred into liquid nitrogen –170°C and kept until use.

For the assays, thawed PBMCs were washed and resuspended. 3x10^6^ PBMCs were used to study B- and T-cell immune-phenotype. The following fluorochromes were used for the B cells: LIVE/DEAD® Fixable Violet Dead Cell Stain (Invitrogen), anti-CD19-AlexaFluor780 (eBioscience), anti-CD20-AlexaFluor700 (eBioscience), anti-CD27-APC (eBioscience), anti-IgD-PE (BD), anti-IgM-PercP-Cy5. 5 (BD), anti-CD24-FITC (eBioscience) and anti-CD38-PECy7 (eBioscience). For other lymphocyte subsets the antibody-dye combinations were as follows: LIVE/DEAD® Aqua Dead Cell Stain (Invitrogen), anti-CD3-FITC (Invitrogen), anti-CD20-AlexaFluor700 (eBioscience), anti-CD56-APC (eBioscience), anti-CD16-eFluor450 (eBioscience ). PBMCs stained with fluorochromes were incubated for 30 minutes at 4°C. Cells were acquired on LSRFortessa (BD). Data were analysed using DIVA software (BD). Gating strategy to identify transitional B cells has been included in Supplementary Figure 2.

*RNA isolation, cDNA synthesis and RT-qPCR*

Peripheral vein blood was drawn directly into Tempus™ Blood RNA Tubes (Life-Technologies) and stored at -20ºC. This standardized sample collection aimed to decrease gene-expression variance. Whole blood RNA was extracted using Tempus™ Spin RNA Isolation (Life-Technologies) and 1. 3 µg of total RNA was reverse transcribed to cDNA using the TaqMan® Reverse Transcription Reagents (Life Technologies) according to manufacturer’s instructions. Thirty ng of the first-strand cDNA obtained was amplified in duplicates for 45 cycles in 20 µl reaction consisting of TaqMan® Universal PCR Master Mix (Life Technologies), 320 nM of each primer and 127. 5 nM of probes of custom assays or inventoried assays in 384-well plates using the 7900HT Real-Time PCR System (Applied Biosystems, Foster City, CA) in the 9600 emulation mode. The details of the assays are in Supplementary Tables 2 and 3. These assays were not detecting genomic DNA and were assessed regularly by no reverse transcription controls. The sequence detector SDS 2. 4 software (Life Technologies) was used to determine the Ct values using a common threshold set at 0. 1 (2) and exported using RQ Manager 1. 2. 1 (Life-Technologies). Prior to the study we confirmed that the target gene expression was optimal, stable over 5 days before freezing and stable after months of freezing (data not shown). We have also introduced a single control sample of peripheral blood, collected at the beginning of the study, in all RT-qPCR and Fluidigm plates to ensure consistency of expression.

*Fluidigm platform.* The expression levels of a set of target genes and 3 endogenous reference genes were measured in 470 RNA samples on the Fluidigm BioMark qPCR platform with a pre-amplification step. The RNA samples from above were treated with TURBO™ DNase (Life-Technologies) to remove genomic DNA, reverse-transcribed as above and pre-amplified using specific primers/assays for 14 cycles. Forty cycles of PCR were performed, using the microfluidic chip developed by BioMark and according to the manufacturer’s instructions.

*Statistical Analyses*

For comparison of baseline demographics, clinical characteristics and predicted probabilities of tolerance across groups, we used chi-squared test or fisher’s exact test as appropriate for binary variables, independent samples t-test or Wilcoxon sign-rank test as appropriate for continuous measures (paired samples test for comparisons between samples from the same patient).

Associations of gene expression or of predicted probability of tolerance with IS drug intake were examined in linear regression models adjusting simultaneously for confounding by IS drugs other than the drug of interest. Three groups of drugs were examined: Prednisone/Prednisolone, CNI (Ciclosporin and Tacrolimus) and antiproliferative drugs (Azathioprine and MMF). Bonferroni correction was applied for multiple comparisons of patients on and off different drugs.

Areas under the ROC curve (AUC) were calculated and compared with the *pROC* package in R (3). Adjusted AUC was derived after fitting a linear regression model of the estimated probability of tolerance on drug regimen in IS patients and calculating the AUC for the residuals of this model (4).

Pre-processing of RT-qPCR and Fluidigm data

RT-qPCR data were pre-processed using the *HTqPCR* package in R (5). Expression values above 35, and those with duplicates outside the 80% quantile interval of the distribution corresponding to their mean and standard deviation were removed. Ct values were normalized using DCt with respect to HPRT (this being the most stable housekeeping gene when compared to ABL, and B2M). Statistical analyses were performed with log_2_ (2^-DCt^) values. The SLC8A1 gene, part of the original IoT signature, was excluded due to being undetected in 36% of the samples. The expression of FoxP3 and Alpha-Mannosidase was measured by RT-qPCR, and their ratio (FoxP3/Alpha-Mannosidase) was added to the multivariate predictor. Fluidigm expression data was pre-processed as above, with the exception of duplicate variation filtering. Data for genes with call rate ≤80% and those genes with complete absence of replication in any sample was removed. The remaining gene list is shown in Supplementary Table 3. The mean coefficient of variation (CV) for all genes retained in the study after QC was 3.5% (SD 1. 6%, range 0.9% - 9.0%) except for PDE4B (CV 19%). The CVs for the 9 genes in the most successful IS-free signature were between 1. 1% and 4. 6%. Outliers were detected gene by gene using a Bonferroni outlier test and recoded to the next highest or lowest value.

Re-analysis of RISET 2. 0 Agilent custom microarray from the IoT study

Details of the RISET 2. 0 array and pre-processing were published elsewhere (1). Probes with non-significant expression above background in more than 20% of the samples were filtered-out, leaving 1,989 out of 5,066 probes for feature selection analyses. Missing gene-expression data were imputed using the k-nearest neighbors method implemented in the R package *Impute* (6). The array-wide analysis of drug effects was carried out using empirical Bayes moderated linear models implemented in the Limma package (7). We estimated the effects of three factors: CNI: (ciclosporin, tacrolimus, none), MMF/Aza (mycophenolate mofetil, azathioprine, none), and prednisone (on steroids, off steroids). Benjamini Hochberg (BH) method was used for multiple testing correction. Genes with an adjusted p-value under 0. 05 were selected as statistically significant. The model represented in equation 1, for the *i^th^* patient, and the *j^th^* gene*,* was estimated using data from stable and chronic rejector patients. The resulting estimated residual ε*_ij_* represents the IS-independent gene expression (IS-IE). Subsequently, the gene-expression of tolerant patients and healthy controls was re-scaled to IS-IE by subtracting the intercept *a_j_* from the raw expression.

 (Eq 1)

To perform the calculation of the drug effects, we chose a simple strategy that distinguished only the absence or the presence of each drug. We would argue that this is the better approach for clinical application, owing to the simplicity of the calculation that has proven to be successful but also, because we do not know the specific immune effect of the different doses in all of the leucocyte subsets, or the shape of the dose-response relationships of each IS drug with gene-expression. Attempting more complex modelling would threaten the reliability and generalizability of the model.

We used the regularized multivariate logistic regression method Elastic-Net implemented in the GLMNET package (8), to select an optimal set of genes predictive of tolerance, comparing the estimated IS-IE in tolerant vs. patients on IS. Classification cut-offs were selected to ensure specificity above 0. 85, while retaining sensitivity above 0. 70. Model parameters were tuned using leave-group-out cross validation with 65% training-set, and 100 resampling iterations, with the Area Under the Curve as accuracy measure, via the *caret* package in R (9). Prior to model estimation, missing values were imputed using K-nearest-neighbors. Genes for which expression was not significantly above background (p>0. 01) in at least 80% of the samples were filtered out prior to analysis (3,081 out of 5,070 probes) to increase statistical power (10). For the identification of differentially expressed biological pathways, we carried out Gene-set analysis (11), using the curated-list of gene-sets from the Broad Institute ([www. broadinstitute. org/gsea](http://www.broadinstitute.org/gsea)). Gene-sets with an associated FDR below 10% were considered differentially expressed.

Validation of the novel signature on the Fluidigm platform using samples from the GAMBIT cohorts

IS-IE was estimated as in the IoT cohort, missing values imputed using K-nearest-neighbors, and outliers were detected and recoded as above. The same machine learning methods based on Elastic-Net were used to select a validated gene-list using as input the 28 genes selected by GLMNET in the IoT cohort. We performed 100 Elastic Net model-selection repeats based on Fluidigm expression of the validated genes and selected the most parsimonious gene-expression signature. In order to test the stability of the signatures, IS-IE from the timepoint 2 samples was fed into the models estimated from timepoint 1 samples, and classification accuracy evaluated with the same cut-off.

**Supplementary Results**

*Selection of the 9-gene signature*

We could validate in the RT-PCR-based Fluidigm platform 26 out of the 28 array genes selected by Elastic Net in the IoT cohort. It became clear from the 100 model-selection repeats that two nested sets of genes emerged: 19 genes were included in the most frequently selected model (69/100) (alpha 0.1, lambda 0.1) and 9 of these genes were included in the next frequently selected model (9/100) reported as the FINAL model (alpha 0.432, lambda 0.1). Of the 26 validated genes 7 were consistently ignored by Elastic Net in all 100 model-selection repeats (CD79B, CHCHD7, CISH, POLR2D, SEC24D, SRGAP2 and TSC22D3).

The performance of the 19-gene model was only marginally better that the 9-gene model (AUC 0.953 (95% CI 0.896 – 1) compared to 0.933 (0.862 – 1), p=0.058 from DeLong test). Consequently, the 9-gene model was selected as the FINAL, because it was more parsimonious and its performance showed no material difference to that of the 19-gene model.

*Important features of the new signature. Different patients are chosen with the new IS-independent signature.*

The clinical use of these signatures is to identify patients on IS that are identified as “possible tolerant” by the chosen signature. Arguably, an important safety issue follows, if the signature is confounded by IS when patients are weaned off they will lose the expression of the signature but not because the possible “tolerance state” has been eliminated, but because the IS effect is now non-existent. This makes impossible to evaluate the underlying mechanism of expression. Further, using an IS-confounded signature would choose the wrong patients for IS weaning. We therefore compared the patients chosen as “possible tolerant” with the IoT signature and the IS-independent signature (Supplementary Table 6) within the GAMBIT cohort. Only 2 patients were positive for both signatures (<10% of the positives). Patients chosen have significant differences in the proportion of them taking mycophenolate, azathioprine and steroids.

*Important features of the new signature. Studying the effect of drugs and time since transplantation.*

The prediction of tolerance based on the IoT signature discriminated against azathioprine treatment, while the prediction of tolerance based on the IS-free signature had a slight bias against MMF treatment that did not reach statistical significance. As patients classified as tolerant according to the IS-free signature had longer times post transplantation than patients classified as tolerant according to the IoT signature (p=0. 029), the association between tolerance prediction and type of drug was re-examined before and after adjustment for time post transplantation (Supplementary Table 7). This adjustment did not influence the results for both signatures.

There is very strong evidence for association between type of IS-drug and time since transplantation for all immunosuppressants but steroids. This is in agreement with the trends in clinical practice where different drugs have been given as standard in different eras, whereas steroids have been used all the time (Supplementary Table 7).

There was no evidence for association between eGFR and type of IS drug, as would be expected for patients with stable kidney function (Supplementary Table 7).

*Important features of the new signature. Estimated probabilities of tolerance in healthy controls and tolerant patients compared to the cut-off*

The estimated probabilities of tolerance for the Tolerant patients were closer to the cut-off when based on the IoT signature but were higher when based on the IS-free signature. In contrast, healthy controls had estimated probabilities significantly higher than the cut-off when based on the IoT signature, but were evenly distributed around the cut-off when based on the IS-free signature. . Correspondingly, the IoT signature classified more healthy controls as tolerant (Supplementary Table 8). Given that it has been demonstrated that operational tolerance is an active process associated with activation of various sub-types of cells of the immune system, it is reasonable to expect that expression related to an immune response in healthy controls would be closer to a neutral mid-point between tolerant and non-tolerant patients, as reflected in the IS-free signature.

*Donor Specific Antibodies from Tolerant recipients.*

After providing the study samples, measurable donor specific antibodies (DSA) were found in 3 tolerant patients, contrary to what was found the IoT study where none of the tolerant recipients had detectable DSA in serum. Two of these patients maintained stable kidney function (one became DSA negative, no further samples were available for the other) and one was re-introduced on immunosuppression 9 months after the last sample, due to a biopsy-for-cause showing borderline changes suspicious of T-cell rejection. However, none of these three patients were outliers regarding the estimated probability of tolerance or the percentages of transitional B-cells. Removing these patients from the analysis did not change the interpretation of the results and they were included in the present study as tolerant at the time of sample collection.

**Supplementary Table 1**: Analysis of drug effects on gene expression measured on the RISET 2.0 microarray platform on samples from the IoT study.

Gene Name: Probe anotation

Gene ID: Gene ID

Log Fold Change: estimate of the log2-fold-change of expression between the groups specified in the last column

Conf Interval L: left limit of the confidence interval for the log2-fold-change

Conf Interval R: right limit of the confidence interval for the log2-fold-change

Adjusted p-value: adjusted p-values found using an optimized false discovery rate (FDR) approach (q-value)

B: log of the odds that the gene is differentially expressed between the groups specified in the last column

Drug Group Comparison: Drug-groups where the expression was being compared.

“None” means absence of that drug vs “MMF” presence of the drug. Drug-groups that had no genes differentially expressed such as “CyA vs Tac” are not shown.

**Supplementary Table 2**: Distribution of drug regimen in stable patients

| CNI | Azathioprine vs MMF | Prednisone | N | Percentage # |
| --- | --- | --- | --- | --- |
| ciclosporin | azathioprine | No | 25 | 13. 2 |
| ciclosporin | azathioprine | Yes | 7 | 3. 7 |
| ciclosporin | MMF | No | 29 | 15. 3 |
| ciclosporin | MMF | Yes | 12 | 6. 3 |
| ciclosporin | None | No | 9 | 4. 7 |
| ciclosporin | None | Yes | 6 | 3. 2 |
| tacrolimus | azathioprine | No | 11 | 5. 8 |
| tacrolimus | azathioprine | Yes | 1 | 0. 5 |
| tacrolimus | MMF | No | 24 | 12. 6 |
| tacrolimus | MMF | Yes | 8 | 4. 2 |
| tacrolimus | None | No | 3 | 1. 6 |
| tacrolimus | None | Yes | 4 | 2. 1 |
| None | azathioprine | No | 1 | 0. 5 |
| None | azathioprine | Yes | 17 | 8. 9 |
| None | MMF | Yes | 12 | 6. 3 |
| None | None | Yes | 12 | 6. 3 |
| None | None | No | 1 * | 0. 5 |

# Percentage from all stable patients

* Patient on Sirolimus single therapy

Note: Information of drug regimen was missing for 8 of the stable patients (4. 2%)

**Supplementary Table 3.**  Detailed clinical description of GAMBIT tolerant patients

|  | Age | Sex | Ys Post-Tx*^a^* | eGFR*^b^* | CRT*^c^* | Lymph*^d^* | Donor Type | HLA-MM*^e^* | Ys IS free*^f^* | Country |
| --- | --- | --- | --- | --- | --- | --- | --- | --- | --- | --- |
| 1 | 44 | Male | 20 | 68. 5 | 108 | 1. 500 | Living | 0, 0, 0 | 7 - 13 | UK |
| 2 | 69 | Male | 13 | 58. 7 | 114 | 1. 000 | Living | 0, 0, 0 | 3. 5 | UK |
| 3 | 77 | Male | 27 | 50. 6 | 127 | 1. 500 | Deceased | 1, 1, 1 | 6 | Spain |
| 4 | 37 | Male | 11 | 68. 7 | 111 | 1. 500 | Deceased | 1, 2, 2 | 2 | UK |
| 5 | 61 | Male | 2 | 67. 7 | 103 | 1. 700 | Living | 0, 0, 0 | 2. 3 | UK |
| 6 | 55 | Male | 31 | - | - | - | Deceased | 1, 0, - | 5. 6 | UK |
| 7 | 33 | Male | 11 | 29. 0 | 239 | 2. 000 | Deceased | 1, 2, 1 | 2. 5 | UK |
| 8 | 52 | Male | 30 | - | - | - | Living | 0, 0, 0 | 5 | UK |
| 9 | 51 | Male | 25 | 60. 6 | 117 | 2. 000 | Deceased | - | 7 | UK |
| 10 | 63 | Female | 24 | 40. 3 | 124 | 2. 200 | Living | 1, 1, 1 | 22. 5 | UK |
| 11 | 41 | Female | 22 | - | 72 | - | Deceased | 1, 2, 0 | 1 | UK |
| 12 | 22 | Male | 11 | 77. 2 | 110 | 3. 200 | Living | 1, 1, 1 | 5 | UK |
| 13 | 35 | Female | 15 | 49. 0 | 116 | - | Deceased | 1, 1, 1 | 1 | UK |
| 14 | 43 | Male | 12 | 67. 4 | 110 | - | Living | 1, 1, 0 | 9. 1 | Sweden |
| Means | 49 | 21% F | 18 | 58 | 121 | 1. 844 | 50% | 1, 1, 0 |  |  |

IS: Immunosuppression. *a*: years between transplant and first sample; *b*: glomerular filtration rate in mL/min; *c*: serum creatinine values in μmol/L; *d*: Lymphocyte counts in peripheral blood cells*10^9^/ dL; *e*: HLA mismatches: number of mismatches at the A locus, at the B locus, at the DR locus; *f*: approximately the number of years between the patient stopping the last immunosuppressant and the first sample obtained for the study, please note for self – withdrawal strategies “stopping date” is very difficult to ascertain.

**Supplementary Table 4.**  Assay list used for the RT-PCR test of the Indices of Tolerance gene-list

| **Gene Symbol** | **Inventoried Assay ID - Primers/ Probes sequences** | **Gene Name** | **R^2^** |
| --- | --- | --- | --- |
| B2M | 5’-GAG TAT GCC TGC CGT GTG-3’ (FWD)  5’- AAT CCA AAT GCG GCA TCT-3’ (REV)  6FAM-CCT CCA TGA TGC TGC TTA CAT GTC TC-TAMRA | Beta-2-microglobulin | 1. 9 |
| CD79B * | Hs00236881_m1 | CD79b molecule, immunoglobulin-associated beta | 9. 8 |
| FCRL1 * | Hs00364705_m1 | Fc receptor-like 1, CD307a | 16. 7 |
| FCRL2 * | Hs00229156_m1 | Fc receptor-like 2, CD307b | 8. 2 |
| FoxP3 | 5’-AAG TGG CCC GGA TGT GAG A-3’ (FWD)  5’-CAT TGT GCC CTG CCC TTC T -3’ (REV)  6FAM-ACT TCC TCA AGC ACT GCC AGG CGG -–TAMRA | Forkhead box P3 | 2. 1 |
| H3ST1 | Hs01099196_m1 | Heparin sulfate (glucosamine) 3-O-sulfotransferase 1 | - |
| MAN1A2, ALG11 | 5’- CGG CCA GAA GTT ATG GAG ACT TAC -3’ (FWD)  5’- CTT AGG CCT GAA TAG CCT CCA TTC -3’ (REV)  6FAM-TGG GAA GCC GTA GAG GCC TTG GAA A -–TAMRA | α-1,2-mannosidase | 2. 2 |
| MS4A1 * | Hs00544818_m1 | Membrane-spanning 4-domains, subfamily A, member 1, CD20, | 10. 0 |
| PNOC | Hs00173823_m1 | Prepronociceptin | 4. 3 |
| SH2D1B * | Hs01592483_m1 | SH2 domain containing 1B | 18. 1 |
| TCL1A | Hs00172040_m1 | T-cell leukemia/lymphoma 1A | 27. 2 |
| TLR5 * | Hs00152825_m1 | Toll-like receptor 5 | 5. 2 |
| CD3Zeta | 5’- TGC TGG ATG GAA TCC TCT TCA T -3’ (FWD)  5’- GGT TCT GGC CCT GCT GGT A -3’ (REV)  6FAM-TGA GAG TGA AGT TCA GCA GGA GCG CA-TAMRA | CD3Zeta, CD247 | 3. 1 |
| HPRT | 5’-AGT CTG GCT TAT ATC CAA CAC TTC G -3’ (FWD)  5’ –GAC TTT GCT TTC CTT GGT CAG G-3’ (REV)  6FAM-TTT CAC CAG CAA GCT TGC GAC CTT GA -–TAMRA | Hypoxanthine phosphoribosyltransferase | - |

* Genes preferentially expressed in B lymphocytes

R^2^ estimates from linear regression models used to adjust each gene for IS drug administration showing the percentage of variance explained by drugs.

**Supplementary Table 5** Gene-list (reliable signal) with assay description used in the Fluidigm platform for elastic net selection of the immunosuppression-free signature.

| **Source** | **Gene Symbol** | **Assay ID** | **Gene Name** | **R^2^** |
| --- | --- | --- | --- | --- |
| GLMNET | AP5S1 | Hs00217922_m1 | Adaptor-related protein complex 5, sigma 1 subunit, C20ORF29 | 1. 8 |
| GLMNET | ATXN3 ‡ | Hs01026447_m1 | Ataxin 3 | 2. 2 |
| GLMNET | BCL2A1 ‡ | Hs00187845_m1 | BCL2-related protein A1 | 2. 1 |
| IoT, GLMNET | CD79B | Hs00236881_m1 | CD79b molecule, immunoglobulin-associated beta | 9. 8 |
| GLMNET | CHCHD7 | Hs02387393_m1 | coiled-coil-helix-coiled-coil-helix domain containing 7 | 1. 6 |
| GLMNET, GMCSF | CISH | Hs00367082_g1 | cytokine inducible SH2-containing protein | 0. 4 |
| GLMNET | DNMT3A | Hs01027166_m1 | DNA (cytosine-5-)-methyltransferase 3 alpha | 2. 6 |
| GLMNET | EBI2, GPR183 | Hs00953886_m1 | EBV-Induced G-Protein Coupled Receptor 2, G Protein-Coupled Receptor 183 | 4. 9 |
| GLMNET | EEF1A1; ‡  EEF1AL7 | Hs00265885_g1 | eukaryotic translation elongation factor 1 alpha 1 | 7 |
| GLMNET | GEMIN7 ‡ | Hs00226769_m1 | gem (nuclear organelle) associated protein 7 | 1. 3 |
| GLMNET | HP | Hs00978377_m1 | haptoglobin | 2. 3 |
| GLMNET | IGHM | Hs00941538_g1 | immunoglobulin heavy constant mu | 11. 4 |
| GLMNET | IGLC1 ‡ | Hs00760769_s1 | immunoglobulin lambda constant 1 (Mcg marker) | 9. 9 |
| GLMNET | IRF2 | Hs01082884_m1 | interferon regulatory factor 2 | 2 |
| GLMNET | MS4A4A ‡ | Hs01106863_m1 | membrane-spanning 4-domains, subfamily A, member 4A | 1. 4 |
| GLMNET, CD40 | NFKBIA ‡ | Hs00153283_m1 | nuclear factor of kappa light polypeptide gene enhancer in B-cells inhibitor, alpha | 6. 2 |
| GLMNET | PDE4B | Hs00963641_m1 | phosphodiesterase 4B, cAMP-specific | 3. 7 |
| GLMNET | POLR2D | Hs00953322_g1 | polymerase (RNA) II (DNA directed) polypeptide D | 2. 4 |
| GLMNET | PPIL3 | Hs00368985_m1 | peptidylprolyl isomerase (cyclophilin)-like 3 | 1. 3 |
| GLMNET | RAB40C ‡ | Hs00368350_m1 | RAB40C, member RAS oncogene family | 3. 8 |
| GLMNET, TNF | RELB | Hs00232399_m1 | v-rel avian reticuloendotheliosis viral oncogene homolog B | 2. 4 |
| GLMNET | SEC24D | Hs00914074_m1 | SEC24 family member D | 0. 8 |
| GLMNET | SRGAP2 | Hs00418582_m1 | pseudogene | 0. 6 |
| GLMNET | TNFAIP1 | Hs01108157_g1 | tumor necrosis factor, alpha-induced protein 1 (endothelial) | 3. 1 |
| GLMNET, CD40 | TNFAIP3 ‡ | Hs00234713_m1 | tumor necrosis factor, alpha-induced protein 3 | 3. 2 |
| GLMNET | TSC22D3 | Hs00608272_m1 | TSC22 domain family, member 3 | 2. 1 |

‡ Genes selected for the IS-independent signature of tolerance

R^2^ estimates from linear regression models used to adjust each gene for IS drug administration showing the percentage of variance explained by drugs. Note: COL9A3 and ITGB1BP1 failed quality control criteria required to provide a reliable signal in Fluidigm platform.

**Supplementary Table 6:** Clinical and demographic characteristics of stable patients from the GAMBIT study classified as tolerant with the IoT and the IS-IE signatures (classification match only in two patients).

|  | **IoT signature** | **IS-IE signature ^d^** | **Tolerant** |
| --- | --- | --- | --- |
| **N** | 25 | 20 | 14 |
| **%Female** | 60. 0 % | 40. 0 % ^ns^ | 21. 4 % |
| **%Deceased** | 44. 0% | 56. 0% ^ns^ | 50. 0% |
| **Yrs-Post-Tx ^a^** | 11. 3 (4. 7 , 30. 7) | 17. 6 (7. 0 , 33. 0) * | 17. 5 (2. 2 , 30. 8) |
| **Age ^b^** | 50. 5 (11. 3) | 54. 5 (11. 2) ^ns^ | 48. 8 (15. 2) |
| **eGFR ^b^** | 57. 0 (17. 4) | 65. 6 (23. 2) ^ns^ | 57. 9 (14. 4) |
| **n HLA-MM ^c^** | 3 (0 - 5) | 3 (0 - 4) ^ns^ | 3 (0-5) |
| **% DSA** (number) | 0 | 5% (1) | 21. 4% (3) |
| **IS ^e^**  **% on Tac.**  **% on Cyc.**  **% on Aza.**  **% on MMF**  **% on Pred.** | 36 %  52 %  4 %  72 %  24 % | 15 % ^ns^  40 % ^ns^  40 % **  15 % ***  60 % * | -  -  -  -  - |
| **IS Dose ^f^**  **Tac.**  μg/L  **Cyc.** μg/L  **Aza.** mg/day  **MMF** mg/day  **Pred.**  mg/day | 5 (2, 10)  150 (50, 250)  75  1000 (500, 1500)  5. 0 (2. 5, 7. 5) | 2 (2, 4) ^ns^  150 (75, 250) ^ns^  87. 5 (50, 100)  1000 (250, 1000) ^ns^  5. 0 (3. 0, 10. 0) ^ns^ | -  -  -  -  - |
| **WBC x 10^-9 b^** | 6. 4 (1. 6) | 7. 6 (2. 5) ^ns^ | 7. 3 (1. 8) |
| **Lymph x 10^-9 b^** | 1. 7 (0. 5) | 1. 6 (0. 8) ^ns^ | 1. 8 (0. 6) |
| **%T-cells** | 72. 4 (8. 3) | 75. 8 (8. 6) ^ns^ | 59. 3 (17. 1) |
| **%B-cells** | 11. 8 (7. 3) | 9. 3 (9. 5) ^ns^ | 22. 9 (16. 1) |
| **%Transitional B-cells** | 4. 9 (3. 2) | 4. 0 (2. 7) ^ns^ | 8. 7 (2. 6) |

IS: Immunosuppression. Notes: **a**: median (min,max); **b**: mean (sd); **c**: median sum of HLA-A, HLA-B & HLA-DR mismatches (min-max); **d**: statistical significance of the differences between stable patients classified as tolerant with the IoT and with the IS-free signatures; **e**: percentage (%) from the total number of patients classified as tolerant in the group; **f**: Median (min, max) doses/trough levels at recruitment among patients receiving the corresponding drug (Tac: Tacrolimus trough levels, Cyc: ciclosporin A trough levels, Aza: Azathioprine oral dose, MMF: Mycophenolate mofetil oral dose; Pred: Prednisone oral dose).

ns >0. 05, *<0. 05; **<0. 01,*** <0. 001.

**Supplementary Table 7**: Association between IS drugs, and time since transplantation, predicted probability of tolerance according to the IoT and IS-IE signatures and eGFR in stable patients from the GAMBIT study

|  | **p-values for drug effects on Predicted Probability of Tolerance** | | **p-values for drug effects on Predicted Probability of Tolerance** (adjusted for time since Tx) | | **p-values for association between drugs and**  **Time since Transplantation** | **p-values for association between drugs and eGFR** |
| --- | --- | --- | --- | --- | --- | --- |
|  | **IoT** | **IS-free** | **IoT** | **IS-free** |  |  |
| **Pred ^a^** | <0.001 | 0.36 | <0.001 | 0.34 | 0.32 | 0.41 |
| **Cyc ^b^** | 1 | 0.45 | 1 | 0.43 | 0.0052 | 0.077 |
| **Tac ^b^** | 1 | 1 | 1 | 1 | 2.80E-06 | 0.23 |
| **MMF ^c^** | 1 | 0.057 | 1 | 0.058 | 0.015 | 0.9 |
| **Aza ^c^** | <0.001 | 1 | <0.001 | 1 | 0.0033 | 0.15 |

**a.**  p-value of the comparison On/Off Prednisone; **b.**  p-value of the comparison On Ciclosporin or Tacrolimus / Off CNI; **c.**  p-value of the comparison On MMF or Azathioprine / Off anti-proliferative drugs.

Note: p-values for comparisons of stable patients on and off each drug are derived after adjustment in a linear regression model for all other drugs/drug groups. P-values for the Cyc/Tac-group (subgroups No-Cyc/Tac, Cyc and Tac) and for the Aza/MMF-group (subgroups No-Aza/MMF, Aza, MMF) were adjusted for multiple comparisons with Bonferroni correction.

**Supplementary Table 8:** Predicted probability of tolerance in Tolerant patients and Healthy control in relation to the cut-off

| **Predicted Probability of Tolerance** | **mean (std. dev.)** | | **Classified as tolerant** | |
| --- | --- | --- | --- | --- |
|  | **IoT signature** | **IS-free signature** | **IoT signature** | **IS-free signature** |
| **Tolerant patients ^a^** | 0.67 (0.12) ^ns^ | 0.62 (0.10) * | 71.4 % | 84.6 % |
| **Healthy controls ^b^** | 0.72 (0.15) * | 0.54 (0.07) ^ns^ | 75.0 % | 45.5 % |

**a.** comparison in a one-sample t-test with the cut-off for IoT signature of 0.62;

**b.** comparison in a one-sample t-test with the cut-off for IS-free signature of 0.54.


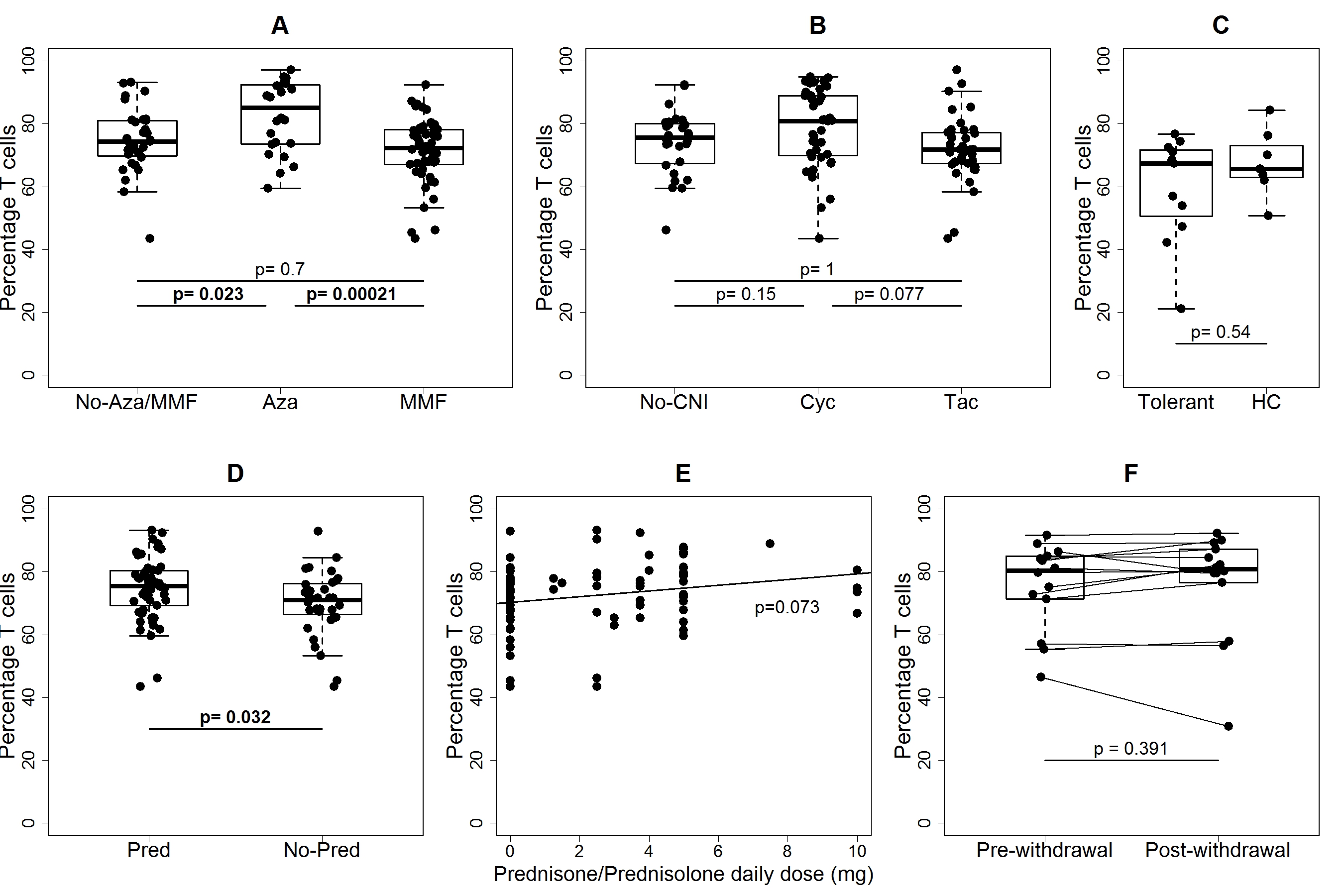


**Supplementary Figure. 1. Percentage of T cells (CD3+ cells within the live lymphocyte gate) in peripheral blood of patients from the GAMBIT cohort.** A) Effect of Anti-proliferative drugs intake in stable patients (n=111) (No-MMF/Aza (n=33), Aza (n=24), MMF (n=54)); B) Effect of CNI drugs intake in stable patients; (n=111) (No-Cyc/Tac (n=28), Cyc (n=41), Tac (n=42)); C) Comparison of Tolerant patients (n=14) and healthy controls (n=12); D) Effect of Prednisone intake in stable patients off azathioprine (n=87) (Pred (n=53), No-Pred (n=34)); E) Effect of Prednisone total daily dose (mg) in stable patients off Azathioprine; F) Comparison of paired samples pre-withdrawal and 3-6 months post-withdrawal from patients who have completed clinically-driven steroid withdrawal (n=16, none receiving azathioprine). P-values for each drug are derived after adjustment in a linear regression model for all other drugs/drug groups. P-values for CNI drugs and for anti-proliferative drugs were adjusted for multiple comparisons with Bonferroni correction. P-values for comparisons between tolerant patients and healthy controls are derived from Wilcoxon test for independent samples. P-values for comparisons pre and post steroid withdrawal are derived from Wilcoxon matched pairs test. Cyc-Ciclosporin, Tac-Tacrolimus, Pred-Prednisone/Prednisolone, Aza-Azathioprine; IS-immunosuppression. HC – Healthy Controls; IoT-Indices of Tolerance study.

**Supplementary Figure 2. Gating strategy for Transitional B cells in flow cytometry.**

B-cells were identified as CD20+CD19+ B-cells. Within the B-cell population, memory B-cells were identified as CD27+ B-cells. IgM+IgD+ cells were gated from the CD27- B-cell population. Within IgM+IgD+ Naive B-cells were identified as CD24+CD38+ cells and transitional B-cells were identified as CD24hiCD38hi cells. Representative dot-plots of total, memory, IgD+IgM+, naïve and transitional B-cells obtained from PBMCs from Tolerant (Tol) and Stable (Sta) patients are shown.


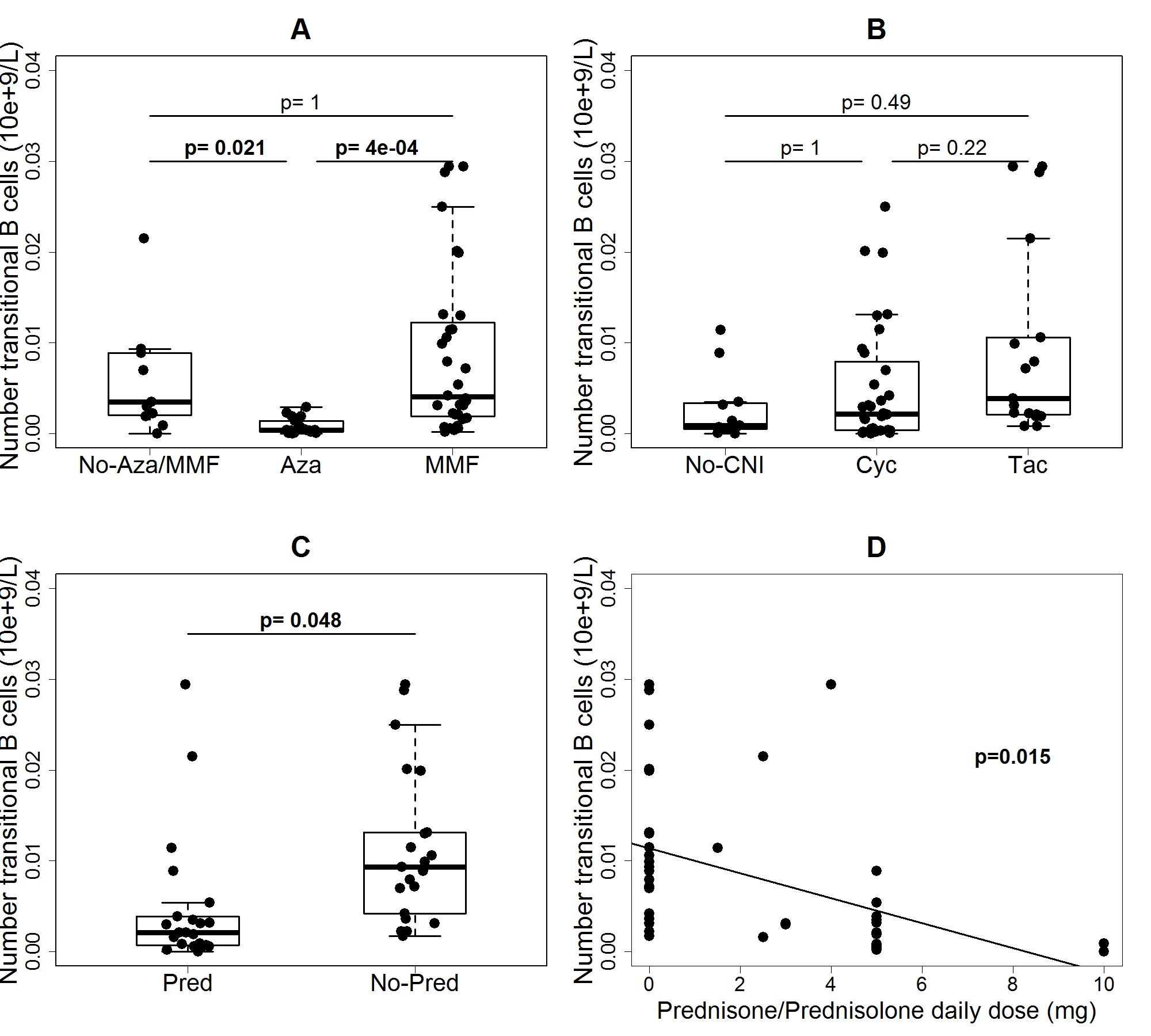


**Supplementary Figure 3. Absolute number of CD24hiCD38hi (transitional B-cells) within the live CD20+CD19+ B lymphocytes and CD27-IgD+IgM+ gate in peripheral blood of patients from the GAMBIT study, cohort 2.** A) Effect of Anti-proliferative drugs intake on transitional B-cell subset size in the stable patients group (n=60) (No-MMF/Aza n=11, Aza n=17, MMF n=32); B) Effect of CNI drugs intake (n=60) (No-Cyc/Tac n=11, Cyc n=32, Tac n=17); C) Effect of prednisone intake on the transitional B-cell subset size in stable patients off azathioprine (n=43) (Pred n=22, No-Pred n=21); D) Effect of Prednisone total daily dose (mg) in stable patients off azathioprine; P-values for each drug are derived after adjustment in a linear regression model for all other drugs/drug groups. P-values for CNI drugs and for anti-proliferative drugs were adjusted for multiple comparisons with Bonferroni correction. Cyc-Ciclosporin, Tac-Tacrolimus, Pred-Prednisone/Prednisolone, Aza-azathioprine; IS-immunosuppression. IoT-Indices of Tolerance study.


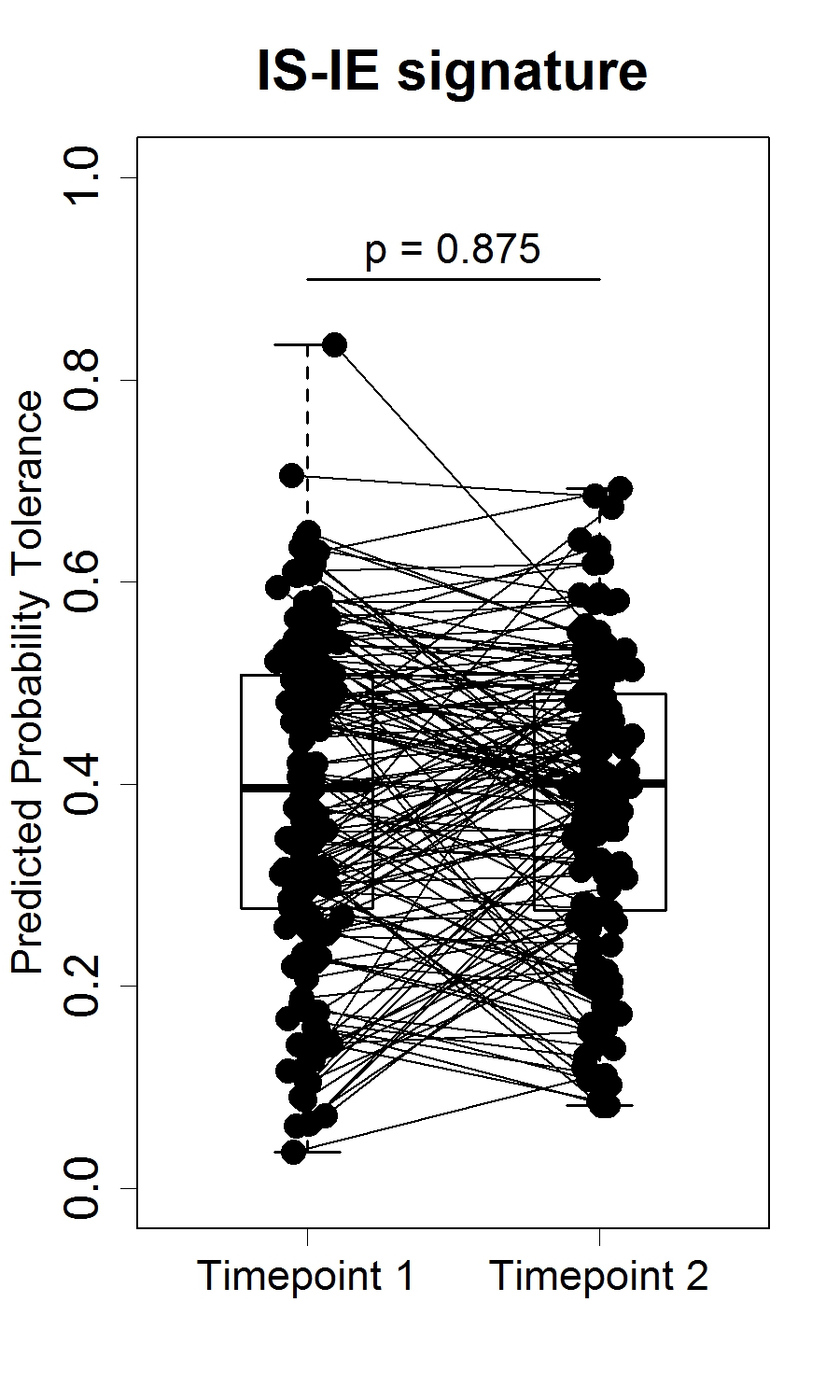
**Supplementary Figure 4. Estimated probability of tolerance is stable over time.**

Based on the new 9-gene-algorithm (gene expression measured in Fluidigm platform in patients from the GAMBIT study, cohorts 2 and 3).

Bibliography for supplementary Materials.

1. Sagoo P, Perucha E, Sawitzki B, Tomiuk S, Stephens DA, Miqueu P et al. Development of a cross-platform biomarker signature to detect renal transplant tolerance in humans. Journal of Clinical Investigation 2010;120(6):1848-1861.

2. Gabert J, Beillard E, van der Velden VH, Bi W, Grimwade D, Pallisgaard N et al. Standardization and quality control studies of 'real-time' quantitative reverse transcriptase polymerase chain reaction of fusion gene transcripts for residual disease detection in leukemia - a Europe Against Cancer program. Leukemia 2003;17(12):2318-2357.

3. Robin X, Turck N, Hainard A, Tiberti N, Lisacek F, Sanchez JC et al. pROC: an open-source package for R and S+ to analyze and compare ROC curves. BMC Bioinformatics 2011;12:77.

4. Janes H, Pepe MS. Adjusting for Covariates in Studies of Diagnostic, Screening, or Prognostic Markers: An Old Concept in a New Setting. American Journal of Epidemiology 2008;168(1):89-97.

5. Dvinge H, Bertone P. HTqPCR: high-throughput analysis and visualization of quantitative real-time PCR data in R. Bioinformatics 2009;25(24):3325-3326.

6. Hastie T, Tibshirani R, Narasimhan B, Chu G. Impute: Imputation for microarray data. R package version 1.38.1. <http://wwwbioconductororg/packages/release/bioc/html/imputehtml> 2014.

7. Smyth GK. Linear models and empirical bayes methods for assessing differential expression in microarray experiments. Stat Appl Genet Mol Biol 2004;3:Article3.

8. Friedman J, Hastie T, Tibshirani R. Regularization Paths for Generalized Linear Models via Coordinate Descent. J Stat Softw 2010;33(1):1-22.

9. Kuhn M. Building Predictive Models in R Using the caret Package. J Stat Softw 2008;28(5):1 - 26.

10. Hackstadt AJ, Hess AM. Filtering for increased power for microarray data analysis. BMC Bioinformatics 2009;10:11.

11. Efron B, Tibshirani R. On Testing the Significance of Sets of Genes. The Annals of Applied Statistics 2007;1(1):107-129.
