# Supplementary material for: Biomarkers of Tolerance in Kidney Transplantation: Are We Predicting Tolerance or Response to Immunosuppressive Treatment?
Source: Am J Transplant. 2016 Aug 8;16(12):3443–57. doi: 10.1111/ajt.13932 (PMC5132071; doi:10.1111/ajt.13932)
Supplement: Supplementary file 2 [file AJT-16-3443-s002.pdf]

Gene Name	Gene ID	log Fold Change	Conf Interval L	Conf Interval R	Adjusted p value	B	Drug Group Comparison
CDCA3	973967	0.8546	0.5031	1.2061	0.0006	7.3356	None vs CyA
SNIP	977359	1.1901	0.8914	1.4888	0.0025	5.4089	None vs CyA
ATP8A1	977475	0.7018	0.2522	1.1514	0.0056	4.1956	None vs CyA
RAB30	973762	1.0627	0.6356	1.4897	0.0056	3.8372	None vs CyA
AP1M2	974318	0.8933	0.6403	1.1462	0.0056	3.7494	None vs CyA
IFNA1	976962	1.1059	0.7779	1.4340	0.0056	3.6972	None vs CyA
NID1	976970	1.2294	0.7858	1.6731	0.0086	3.1772	None vs CyA
JAK3	978079	1.0117	0.7565	1.2670	0.0097	2.9510	None vs CyA
KIFC3	976233	0.9925	0.6002	1.3848	0.0101	2.8042	None vs CyA
CDS1	976925	1.1187	0.7339	1.5034	0.0107	2.6591	None vs CyA
FADS2	975093	0.9469	0.6954	1.1983	0.0113	2.5208	None vs CyA
BG944303	978367	0.8245	0.3097	1.3393	0.0116	2.3347	None vs CyA
NGFR	976625	1.0493	0.6735	1.4251	0.0116	2.2942	None vs CyA
ORAI2	977611	1.1702	0.6822	1.6582	0.0116	2.2810	None vs CyA
PRSS2	978703	0.8541	0.5638	1.1444	0.0131	2.1073	None vs CyA
RAD51L1	978584	1.0599	0.6727	1.4470	0.0146	1.9196	None vs CyA
STAT5B	977130	1.1570	0.8307	1.4834	0.0146	1.8852	None vs CyA
IL28B	974521	1.0172	0.7113	1.3230	0.0146	1.8504	None vs CyA
PDE1A	976924	0.6677	0.1934	1.1419	0.0150	1.7777	None vs CyA
MS4A7	975468	-0.6813	-1.1514	-0.2112	0.0172	1.5969	None vs CyA
CCRL2	977807	0.8514	0.5599	1.1429	0.0172	1.4139	None vs CyA
IL22RA1	977709	1.0100	0.6216	1.3983	0.0172	1.3953	None vs CyA
LOC118945;LOC642413;LOC644496	976698	-1.2123	-1.5252	-0.8994	0.0172	1.3938	None vs CyA
CYBA	974148	0.3924	-0.2399	1.0248	0.0172	1.3640	None vs CyA
AK022418	978635	0.9335	0.4983	1.3686	0.0172	1.3391	None vs CyA
DSG4	974694	0.9365	0.5927	1.2803	0.0172	1.2926	None vs CyA
OR4D1	975031	0.8810	0.5749	1.1871	0.0172	1.2673	None vs CyA
AKAP6	978646	0.9750	0.4320	1.5180	0.0172	1.2465	None vs CyA
CTSL3	974053	-1.0463	-1.3949	-0.6977	0.0172	1.2460	None vs CyA
C17ORF65	977525	0.9791	0.1417	1.8165	0.0172	1.2429	None vs CyA
CD80	974857	0.9266	0.7085	1.1447	0.0174	1.2032	None vs CyA
INHA	977481	0.8163	0.3652	1.2674	0.0185	1.1044	None vs CyA
C10ORF109	976401	1.0797	0.4767	1.6827	0.0185	1.0910	None vs CyA
MATK	973758	0.7156	0.2022	1.2289	0.0193	1.0255	None vs CyA
TRY6	976120	1.0028	0.4051	1.6006	0.0194	0.9837	None vs CyA
RARA	978632	1.3442	0.9587	1.7298	0.0194	0.9707	None vs CyA
DUSP4	978375	0.7649	0.5690	0.9608	0.0212	0.8635	None vs CyA
CD177	976431	0.7614	0.4156	1.1072	0.0223	0.7971	None vs CyA
MRAS	975233	-1.2289	-1.5758	-0.8821	0.0225	0.7663	None vs CyA
HMMR	978086	0.6513	0.1904	1.1123	0.0226	0.7092	None vs CyA
PSMD8	977174	0.9224	0.7054	1.1394	0.0226	0.6978	None vs CyA
PDE7B	975138	-1.0611	-1.5759	-0.5463	0.0226	0.6947	None vs CyA
IL27	977429	0.7010	0.0147	1.3874	0.0249	0.5806	None vs CyA
KCNK7	974407	1.0411	0.7886	1.2937	0.0249	0.5532	None vs CyA
RETNLB	978048	0.7149	0.2995	1.1304	0.0249	0.5452	None vs CyA
CYP1A2	975843	0.6450	-0.0389	1.3289	0.0253	0.4951	None vs CyA
SFTPC	976971	0.7544	0.0291	1.4797	0.0253	0.4719	None vs CyA
NFATC4	974757	0.9392	0.5938	1.2847	0.0253	0.4700	None vs CyA
KCNQ5	978796	0.3940	-0.0919	0.8799	0.0253	0.4544	None vs CyA
FERMT2	977531	1.2124	0.9982	1.4266	0.0253	0.4365	None vs CyA
SCGB1A1	974218	1.2125	0.9446	1.4804	0.0259	0.3987	None vs CyA
TEAD1	977443	0.9948	0.5027	1.4868	0.0262	0.3701	None vs CyA
C12ORF29;CEP290	978511	0.6060	0.2451	0.9669	0.0266	0.3361	None vs CyA
MAPK7	978430	1.0756	0.7814	1.3698	0.0266	0.3220	None vs CyA
PNKD	975121	0.5337	-0.0352	1.1026	0.0267	0.3041	None vs CyA

ACHE	976138	1.0634	0.6354	1.4914	0.0283	0.2188	None vs CyA
NOTCH4	976833	-0.6488	-1.1359	-0.1617	0.0283	0.2132	None vs CyA
IL17C	977449	0.9266	0.4587	1.3945	0.0283	0.2048	None vs CyA
TNFRSF21	977901	-0.4995	-1.1197	0.1206	0.0290	0.1551	None vs CyA
DDN	975153	0.6188	-0.0811	1.3186	0.0290	0.1519	None vs CyA
KMO	977196	0.4248	0.0762	0.7734	0.0301	0.1002	None vs CyA
NAB2	973742	0.8242	0.4103	1.2381	0.0301	0.0670	None vs CyA
HECW1	977045	0.7031	0.4440	0.9623	0.0301	0.0614	None vs CyA
C4ORF26	978227	0.6422	0.2930	0.9913	0.0301	0.0520	None vs CyA
CCL4;CCL4L1;CCL4L2;LOC728835;LOC730424	978414	0.7110	0.3697	1.0523	0.0301	0.0255	None vs CyA
SGIP1	975124	0.7806	0.4193	1.1420	0.0301	0.0088	None vs CyA
EPB41L1	977528	-0.7230	-1.0074	-0.4387	0.0301	-0.0003	None vs CyA
THC2378360	974438	0.9517	0.4235	1.4799	0.0301	-0.0194	None vs CyA
AW242604	975262	0.6500	0.3087	0.9913	0.0301	-0.0679	None vs CyA
DEFA4	978047	1.3303	0.9371	1.7235	0.0301	-0.0719	None vs CyA
THC2271582	975435	0.8381	0.2330	1.4432	0.0301	-0.0824	None vs CyA
OR5AP2	977457	1.2345	0.8453	1.6237	0.0301	-0.0863	None vs CyA
GM2A	975708	0.9138	0.1239	1.7038	0.0301	-0.0928	None vs CyA
STRN4	976597	0.7780	0.3718	1.1841	0.0301	-0.0971	None vs CyA
SRC	974403	-0.6293	-0.8416	-0.4171	0.0301	-0.0981	None vs CyA
KIF15	974944	0.8239	0.5443	1.1035	0.0301	-0.1072	None vs CyA
CSF1R	974483	-0.5907	-1.2146	0.0333	0.0301	-0.1155	None vs CyA
COL12A1	977334	1.2047	0.7412	1.6683	0.0301	-0.1171	None vs CyA
LAMB1	976351	1.2636	0.9378	1.5895	0.0346	-0.2505	None vs CyA
DGCR8	974007	0.7434	0.4216	1.0652	0.0355	-0.2927	None vs CyA
THC2423232	977753	0.9798	0.3683	1.5912	0.0355	-0.2974	None vs CyA
SYNGR3	973985	0.5020	0.1101	0.8940	0.0363	-0.3279	None vs CyA
NOS1	977010	0.7066	0.3903	1.0228	0.0377	-0.3711	None vs CyA
LOC283588	977826	0.8482	0.2278	1.4685	0.0378	-0.3858	None vs CyA
TJP1	975361	0.9690	0.5343	1.4036	0.0414	-0.4945	None vs CyA
NRTN	973959	0.5111	-0.1774	1.1997	0.0414	-0.4967	None vs CyA
SLCO3A1	977485	0.6862	0.2210	1.1514	0.0414	-0.4978	None vs CyA
FTH1	978535	0.7211	0.1981	1.2442	0.0417	-0.5132	None vs CyA
EPS8	975960	-0.9741	-1.3903	-0.5579	0.0423	-0.5363	None vs CyA
CD276	977108	0.7041	0.3557	1.0525	0.0431	-0.5633	None vs CyA
FOXQ1	974539	1.0208	0.4921	1.5495	0.0433	-0.5777	None vs CyA
HGF	978507	-0.7707	-1.1375	-0.4039	0.0437	-0.5952	None vs CyA
MST1	978538	0.7644	0.3045	1.2244	0.0437	-0.6046	None vs CyA
GNAO1	977081	0.8747	0.3451	1.4043	0.0445	-0.6379	None vs CyA
FOXP3	975674	0.7365	-0.0072	1.4801	0.0445	-0.6436	None vs CyA
C11ORF42	978121	0.7695	0.3746	1.1644	0.0445	-0.6483	None vs CyA
TMPRSS2	974714	0.6211	0.2898	0.9524	0.0459	-0.6865	None vs CyA
AXL	974732	0.6585	0.1803	1.1368	0.0459	-0.6952	None vs CyA
APOE	974222	0.6627	0.3635	0.9619	0.0468	-0.7206	None vs CyA
NTRK2	977884	0.4701	0.1600	0.7802	0.0469	-0.7317	None vs CyA
APOA2	978022	0.6167	0.1296	1.1039	0.0475	-0.7588	None vs CyA
CST7	975252	0.7004	0.1959	1.2050	0.0475	-0.7826	None vs CyA
PCP4	974226	0.7934	0.3919	1.1949	0.0475	-0.7969	None vs CyA
CKB	977376	-0.5567	-1.2311	0.1177	0.0475	-0.8028	None vs CyA
GK5	975558	0.6258	0.2233	1.0284	0.0475	-0.8077	None vs CyA
RPS6KL1	977096	0.5705	0.2748	0.8663	0.0475	-0.8199	None vs CyA
HAVCR1	974273	0.7696	0.4153	1.1240	0.0475	-0.8287	None vs CyA
PRSS3	978541	0.8023	0.2337	1.3708	0.0475	-0.8291	None vs CyA
NTF3	978017	0.4824	0.2430	0.7218	0.0475	-0.8328	None vs CyA
A_24_P940041	975794	0.6922	-0.1062	1.4906	0.0475	-0.8345	None vs CyA
TNC	978763	0.5634	0.3674	0.7594	0.0475	-0.8404	None vs CyA
COX7A1	978517	0.6902	0.4646	0.9159	0.0475	-0.8453	None vs CyA
C2	975281	-0.7138	-1.2246	-0.2030	0.0475	-0.8545	None vs CyA

THC2425726	974737	0.8969	0.5058	1.2880	0.0475	-0.8725	None vs CyA
C3ORF23_exon1b	973869	0.3694	-0.1126	0.8514	0.0475	-0.8732	None vs CyA
CPVL	974341	-0.7287	-2.7088	1.2515	0.0475	-0.8752	None vs CyA
A_24_P298179	977777	-0.9290	-1.7505	-0.1074	0.0484	-0.8996	None vs CyA
PTPRO	975699	-0.7314	-1.1311	-0.3317	0.0488	-0.9132	None vs CyA
TNFSF18	976573	-0.9014	-1.5041	-0.2987	0.0251	-0.0546	None vs Tac

KIAA0101	973902	1.7581	1.0307	2.4855	0.0003	7.3446	MMF vs Aza
A_32_P218079	977255	-0.9545	-1.6678	-0.2413	0.0003	7.0325	MMF vs Aza
IGJ	975733	1.6550	0.8268	2.4831	0.0003	6.8784	MMF vs Aza
PTPRN	974149	0.9566	0.4778	1.4354	0.0015	5.2489	MMF vs Aza
IL17RD	975095	-0.5595	-1.1977	0.0788	0.0054	3.8772	MMF vs Aza
STAT1	978150	-0.9968	-1.5059	-0.4877	0.0054	3.7251	MMF vs Aza
HMMR	976055	1.1015	0.6614	1.5415	0.0057	3.5374	MMF vs Aza
CDKN3	974654	1.1019	0.8533	1.3505	0.0078	3.1352	MMF vs Aza
TTC23	977727	-0.9242	-1.2821	-0.5662	0.0110	2.7240	MMF vs Aza
IL5	977756	-0.5125	-0.9739	-0.0510	0.0139	2.4161	MMF vs Aza
KRT26	975947	-0.5324	-0.9190	-0.1457	0.0179	2.0455	MMF vs Aza
PTTG3	976484	0.9108	0.0742	1.7475	0.0179	2.0278	MMF vs Aza
SH2D1B	975146	-1.0810	-1.6952	-0.4667	0.0189	1.9038	MMF vs Aza
RGS16	973950	0.8412	0.5135	1.1690	0.0209	1.7491	MMF vs Aza
NOD1	976835	-0.6414	-1.5369	0.2541	0.0249	1.5301	MMF vs Aza
LINGO2	974698	-1.7721	-2.2656	-1.2786	0.0265	1.4037	MMF vs Aza
LRRC16A	977501	-0.6401	-1.3266	0.0463	0.0265	1.3474	MMF vs Aza
AK096721	978278	-1.0299	-1.4018	-0.6579	0.0265	1.1996	MMF vs Aza
GPBAR1	977388	-0.5832	-0.9695	-0.1969	0.0265	1.1918	MMF vs Aza
RNF213	974264	-0.9926	-1.4209	-0.5643	0.0265	1.1641	MMF vs Aza
SERPING1	977689	-1.4064	-1.9886	-0.8242	0.0265	1.1031	MMF vs Aza
C1ORF101	976793	-0.5254	-1.0208	-0.0299	0.0265	1.0807	MMF vs Aza
ASPM	975384	0.9315	0.3755	1.4874	0.0265	1.0227	MMF vs Aza
IRF7	975459	-1.1206	-1.5911	-0.6501	0.0265	1.0209	MMF vs Aza
C14ORF129	974427	0.6837	0.3933	0.9740	0.0265	1.0125	MMF vs Aza
A_24_P912279	977737	-0.6220	-1.1973	-0.0466	0.0265	0.9682	MMF vs Aza
LOC100130771	974428	-0.6536	-3.0122	1.7050	0.0265	0.9465	MMF vs Aza
CCNG1	977360	0.7103	0.2240	1.1967	0.0294	0.8217	MMF vs Aza
CLIC3	975067	-0.9421	-1.8330	-0.0512	0.0305	0.7550	MMF vs Aza
THC2280863	978567	-0.8000	-1.1815	-0.4184	0.0305	0.7144	MMF vs Aza
CDC2	973843	0.9918	0.6237	1.3599	0.0305	0.6825	MMF vs Aza
LY6E	977685	-1.1636	-1.6390	-0.6881	0.0305	0.6703	MMF vs Aza
AK091192	975320	-0.6241	-1.0437	-0.2046	0.0306	0.6127	MMF vs Aza
PITX2	978772	-1.3075	-1.7407	-0.8743	0.0306	0.5790	MMF vs Aza
APOL2	976040	-0.7812	-1.0793	-0.4831	0.0306	0.5480	MMF vs Aza
AF086115	974696	-0.3934	-0.6888	-0.0981	0.0306	0.5414	MMF vs Aza
CCNB2	977330	1.0638	0.5974	1.5303	0.0306	0.5376	MMF vs Aza
ENST00000322032	975212	2.2739	1.6707	2.8772	0.0318	0.4816	MMF vs Aza
MGC24103	977418	-1.8242	-2.3314	-1.3171	0.0324	0.4409	MMF vs Aza
FSTL5	977688	-0.5786	-1.0414	-0.1157	0.0330	0.4002	MMF vs Aza
ULK4	977957	-0.9618	-1.3407	-0.5829	0.0331	0.3339	MMF vs Aza
RGS5	975012	-0.4344	-0.7893	-0.0795	0.0331	0.3144	MMF vs Aza
AI161251	976803	-1.0877	-1.7790	-0.3964	0.0331	0.3075	MMF vs Aza
SAMD9L	975703	-0.8568	-1.1157	-0.5980	0.0331	0.3066	MMF vs Aza
SP100	974637	-0.7044	-1.1915	-0.2172	0.0331	0.2936	MMF vs Aza
IGHA1	978701	1.1431	0.6623	1.6240	0.0337	0.2511	MMF vs Aza
RABGGTB	976531	0.8820	0.4773	1.2867	0.0337	0.2391	MMF vs Aza
ATP6V0D2	978341	-0.6293	-1.0418	-0.2167	0.0373	0.1308	MMF vs Aza
C9ORF91	975569	-0.6686	-1.0839	-0.2533	0.0374	0.1095	MMF vs Aza
C2	975281	-0.8988	-1.7617	-0.0360	0.0381	0.0648	MMF vs Aza
LOC342892	973824	-0.5997	-1.3525	0.1531	0.0381	0.0566	MMF vs Aza
IFITM2	975409	-0.6812	-1.4960	0.1336	0.0382	0.0382	MMF vs Aza

FRMD4A	975166	-0.3979	-0.8035	0.0078	0.0386	0.0110	MMF vs Aza
LAP3	977125	-0.8573	-1.5851	-0.1295	0.0409	-0.0563	MMF vs Aza
MAN1A1	973922	1.0808	0.6594	1.5022	0.0433	-0.1639	MMF vs Aza
ZNF618	975894	-0.9874	-1.3217	-0.6530	0.0433	-0.1678	MMF vs Aza
UGT2A3	978249	-0.4697	-1.0927	0.1534	0.0433	-0.1801	MMF vs Aza
KIAA1618	976543	-0.7845	-1.3804	-0.1885	0.0433	-0.1841	MMF vs Aza
IGHG1;IGHG2;IGHG3;IGHG4	977160	1.6929	1.2811	2.1048	0.0433	-0.2104	MMF vs Aza
ZBP1	977269	-0.9318	-1.4290	-0.4347	0.0433	-0.2106	MMF vs Aza
HS3ST2	978153	-0.7160	-1.0806	-0.3515	0.0433	-0.2210	MMF vs Aza
LOC100132941	976525	1.2253	0.7599	1.6908	0.0433	-0.2357	MMF vs Aza
GLDC	978695	0.9896	0.6009	1.3783	0.0433	-0.2490	MMF vs Aza
IGKC	976931	1.1042	0.6823	1.5261	0.0433	-0.2586	MMF vs Aza
ENST00000359488	978063	1.6334	0.7906	2.4762	0.0460	-0.3259	MMF vs Aza
UBE2Q2	978596	0.8516	0.5797	1.1235	0.0463	-0.3448	MMF vs Aza
A_24_P926337	976868	-0.5102	-0.9902	-0.0302	0.0465	-0.3750	MMF vs Aza
ACACA	973851	-0.4742	-0.8921	-0.0563	0.0465	-0.3762	MMF vs Aza
EGF	974980	0.8560	0.2191	1.4928	0.0474	-0.4142	MMF vs Aza
GBP2	976470	-0.7636	-1.7416	0.2143	0.0474	-0.4283	MMF vs Aza
C1ORF194;LOC642399	977232	-0.7323	-1.2226	-0.2419	0.0474	-0.4309	MMF vs Aza
HJURP	976828	0.7579	0.1889	1.3269	0.0475	-0.4458	MMF vs Aza
CNIH	974032	0.5809	0.1908	0.9709	0.0475	-0.4664	MMF vs Aza
TUBE1	977516	0.6770	0.2255	1.1285	0.0475	-0.4850	MMF vs Aza
EPSTI1	976982	-1.2093	-2.1483	-0.2703	0.0475	-0.4895	MMF vs Aza
ARSJ	976278	-0.5069	-1.1250	0.1111	0.0475	-0.4933	MMF vs Aza
A_24_P626812	976048	0.5728	-0.0944	1.2400	0.0481	-0.5158	MMF vs Aza
C14ORF79	978794	-0.6499	-1.1966	-0.1032	0.0482	-0.5293	MMF vs Aza
FGD2	974066	-0.7087	-1.0641	-0.3533	0.0484	-0.5442	MMF vs Aza
HPGD	978195	1.0427	0.7548	1.3307	0.0496	-0.5757	MMF vs Aza
MAD2L1	976418	0.9245	0.3759	1.4732	0.0496	-0.5877	MMF vs Aza
HMMR	978086	0.9761	0.1688	1.7834	0.0332	3.4774	None vs MMF
PTPRN	974149	0.8772	0.2185	1.5358	0.0472	2.5925	None vs MMF
PDE7B	975138	1.1522	0.6808	1.6236	0.0441	3.0836	None vs Pred
